# Gain-of-function factor H–related 5 protein impairs glomerular complement regulation resulting in kidney damage

**DOI:** 10.1073/pnas.2022722118

**Published:** 2021-03-22

**Authors:** Talat H. Malik, Daniel P. Gitterman, Deborah P. Lavin, Hannah J. Lomax-Browne, E. Christina Hiemeyer, Linda B. Moran, Katharina Boroviak, H. Terence Cook, Alyssa C. Gilmore, Mawj Mandwie, Amina Ahmad, Ian E. Alexander, Grant J. Logan, Kevin J. Marchbank, Allan Bradley, Matthew C. Pickering

**Affiliations:** ^a^Centre for Inflammatory Disease, Imperial College London, London W12 0NN, United Kingdom;; ^b^Wellcome Trust Sanger Institute, Wellcome Genome Campus, Hinxton, Cambridge CB10 1SA, United Kingdom;; ^c^North West London Pathology, Imperial College Healthcare National Health Service Trust, London W6 8RF, United Kingdom;; ^d^Gene Therapy Research Unit, Children’s Medical Research Institute and Sydney Children’s Hospitals Network, The University of Sydney, NSW 2145 Westmead, Australia;; ^e^Discipline of Child and Adolescent Health, Sydney Medical School, Faculty of Medicine and Health, The University of Sydney, NSW 2145 Westmead, Australia;; ^f^Translational and Clinical Research Institute, The Medical School, Newcastle University, Framlington Place, Newcastle-upon-Tyne NE2 4HH, United Kingdom;; ^g^National Renal Complement Therapeutics Centre, Newcastle-upon-Tyne NE1 4LP, United Kingdom

**Keywords:** complement, kidney, immunology

## Abstract

Genetic variation within the factor H–related (FHR) genes is associated with the complement-mediated kidney disease, C3 glomerulopathy (C3G). There is no definitive treatment for C3G, and a significant proportion of patients develop end-stage renal disease. The prototypical example is CFHR5 nephropathy, through which an internal duplication within a single *CFHR5* gene generates a mutant FHR5 protein (FHR5mut) that leads to accumulation of complement C3 within glomeruli. To elucidate how abnormal FHR proteins cause C3G, we modeled CFHR5 nephropathy in mice. Animals lacking the murine factor H (FH) and FHR proteins, but coexpressing human FH and FHR5mut (hFH-FHR5mut), developed glomerular C3 deposition, whereas mice coexpressing human FH with the normal FHR5 protein (hFH-FHR5) did not. Like in patients, the FHR5mut had a dominant gain-of-function effect, and when administered in hFH-FHR5 mice, it triggered C3 deposition. Importantly, adeno-associated virus vector-delivered homodimeric mini-FH, a molecule with superior surface C3 binding compared to FH, reduced glomerular C3 deposition in the presence of the FHR5mut. Our data demonstrate that FHR5mut causes C3G by disrupting the homeostatic regulation of complement within the kidney and is directly pathogenic in C3G. These results support the use of FH-derived molecules with enhanced C3 binding for treating C3G associated with abnormal FHR proteins. They also suggest that targeting FHR5 represents a way to treat complement-mediated kidney injury.

## Significance

The complement system is integral to innate immunity and host defense. However, inappropriate activation causes host tissue damage and disease. In health, this is prevented by a complex protein network that includes the factor H proteins. Understanding control of complement is critical to treat complement-mediated disease. We demonstrate that a gain-of-function mutant factor H–related 5 protein (FHR5) results in glomerular damage. The mutant interfered with complement regulation within the kidney, resulting in complement accumulation within glomeruli and kidney damage. Administration of a complement regulator with enhanced surface regulatory activity reduced mutant-associated glomerular complement. FHR5 can disrupt the homeostatic regulation of complement within the kidney, and targeting FHR5 represents a way to treat some types of complement-mediated kidney injury.

The complement system is an important component of the immune response to pathogens, particularly meningococcal infection. Complement activation is tightly regulated to prevent its effectors from damaging host tissue. Impaired control of activation, termed complement dysregulation, is associated with tissue injury, including age-related macular degeneration and renal disease. Complement-mediated kidney damage is exemplified by thrombotic microangiopathy in atypical hemolytic uraemic syndrome and glomerular damage in C3 glomerulopathy (C3G) and IgA nephropathy (IgAN).

Complement factor H (FH) is a plasma protein that down-regulates C3 activation through the complement alternative pathway. The essential role of FH is illustrated by homozygous FH-deficient patients who have acquired severe C3 deficiency due to uncontrolled C3 consumption (1). The FH protein family includes five factor H–related proteins (FHR1 through 5), and all are composed of subunits called short consensus repeat (SCR) domains. While FH contains both regulatory and binding SCR domains for the activated C3 fragment C3b, the FHR proteins contain only binding domains, suggesting different functions. The importance of FHR proteins in renal pathology is derived from the associations between susceptibility to C3G and abnormal FHR proteins (2–8). C3G is characterized by dominant glomerular C3 deposition and glomerular damage (9). The prototypic example of FHR-associated C3G is CFHR5 nephropathy (3, 10). Affected individuals have a heterozygous internal duplication within the *CFHR5* gene. The normal FHR5 protein consists of 9 SCR domains, whereas the abnormal FHR5 protein (FHR5mut), due to duplicated exons encoding the first two SCR domains, consists of 11 SCR domains. There are now several examples of abnormal FHR proteins and C3G (2, 4–8).

Both FHR1 (11–13) and FHR5 (14, 15) influence susceptibility to IgAN, a glomerular disorder characterized by galactose-deficient IgA1 immune deposits and C3 deposition. How FHR proteins influence glomerular C3 deposition in both C3G and IgAN remains poorly understood. FHR1 and FHR5 proteins can antagonize the ability of FH to down-regulate C3 activation in vitro (7, 16). However, FHR1, FHR4, and FHR5 can also promote C3 activation in vitro independently of FH (17–19). From these in vitro observations, it can be hypothesized that the degree of C3 deposition in response to a complement-activating trigger within the kidney (e.g., IgA1 immune deposits) depends on the relative interactions between local complement activation and either FH (inhibition of activation) or the FHR proteins (promotion of activation). The degree of complexity in this system is also governed by context-specific interactions between the FHR proteins and surface glycans (20). However, the lack of appropriate in vivo models (21) due to interspecies differences in the FHR proteins has prevented researchers from modeling FHR-associated renal pathology to identify mechanisms of injury and therapeutics, an approach that has been very successful for FH-associated renal pathologies (22–30).

To overcome these limitations, we developed murine strains consisting of 1) mice lacking the entire 664 kb FH-FHR locus and therefore deficient in FH and all the FHR proteins (delFH-FHR), 2) mice lacking the 537 kb FHR locus and therefore expressing normal FH but not the FHR proteins (delFHR), and 3) mice expressing human FH (hFH) and FHR5/FHR5mut in the absence of the mouse proteins. Using these unique models, we show that delFH-FHR animals develop a spontaneous C3G, that the absence of the FHR proteins in the delFHR strain did not result in spontaneous renal disease, and that delFH-FHR animals transgenically expressing hFH with the FHR5mut associated with CFHR5 nephropathy, spontaneously develop C3G, recapitulating the key features of the human disease. Finally, we demonstrate that adeno-associated virus (AAV) vector delivery of a homodimeric mini-FH (HDM-FH) molecule, previously shown to be efficacious in reducing glomerular C3 in FH-deficient mice (30), can significantly reduce glomerular C3 in our CFHR5 nephropathy mouse model and, thus, could be a future therapeutic approach for treating C3G associated with abnormal FHR proteins.

## Results

### delFH-FHR Animals Develop Spontaneous C3G, Whereas delFHR Animals Do Not.

**Fig. 1.**
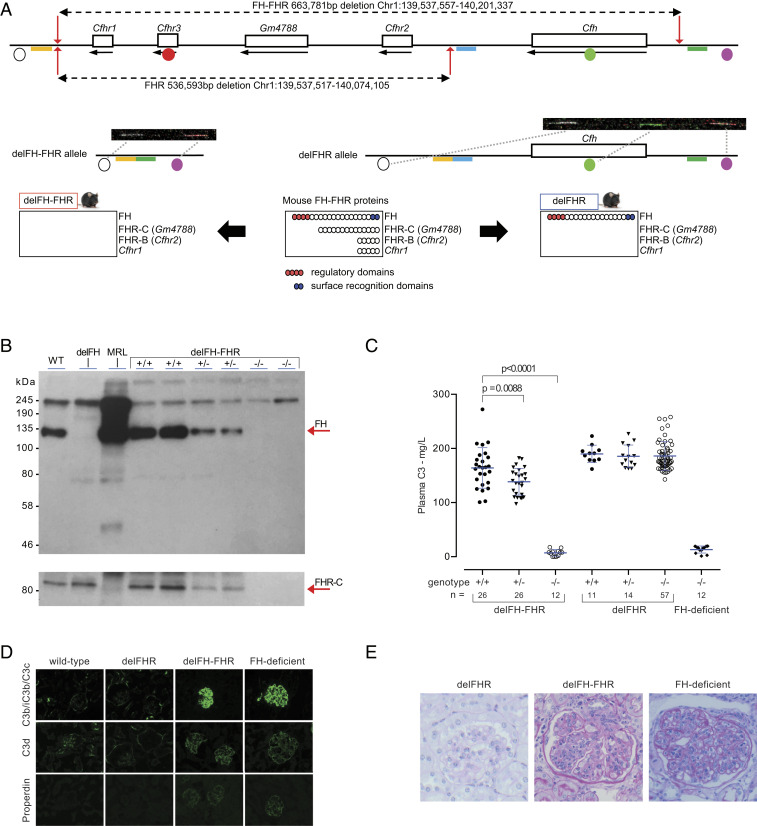
delFH-FHR mice develop spontaneous C3 glomerulopathy, whereas delFHR mice do not. (*A*) Schematic of the mouse FH-FHR locus and proteins depicting the genomic deletions and strains. The delFH-FHR strain is deficient in both FH and the FHR proteins. The delFHR strain is deficient in the FHR proteins only. Fiber-FISH using four probes (circles) across the locus confirmed the deletions. gRNAs (red arrows) and bridging repair oligonucleotides (delFHR: yellow/blue bars; delFH-FHR: yellow/green bars) are indicated, and genome coordinates are derived from Ensembl Mus Musculus version 100.38, Genome Reference Consortium Mouse Build 38 patch release 6. (*B*) Western blot of plasma FH and FHR-C (arrows) in delFH-FHR mice. Control lanes: plasma from wild-type (WT), FH-deficient, and Murphy Roths Large (MRL) (FHR-C–deficient) mice. In the upper blot image, plasma FH is detected with rat anti-mouse FH. In the lower blot image, FHR-C is detected with a sheep anti-mouse FH antibody. FH and FHR-C are reduced and absent in heterozygous and homozygous delFH-FHR mice, respectively. (*C*) Plasma C3 levels in delFH-FHR mice. Bars denote the mean, and whiskers denote the SD. *P* values are derived from ANOVA with Bonferroni multiple comparisons test. Plasma from FH-deficient mice was used as a control. (*D*) Representative glomerular immunostaining images of C3b/iC3b/C3c, C3d, and properdin in WT, delFHR, delFH-FHR, and FH-deficient mice. Abnormal glomerular C3 and properdin staining is evident in the delFH-FHR and FH-deficient mice. (*E*) Representative light microscopic images of glomeruli from 8-mo-old delFH-FHR, delFHR, and age-matched FH-deficient mice. Glomerular hypercelluarity and capillary wall thickening is evident in the delFH-FHR and FH-deficient mice. Glomeruli were normal in the delFHR strain.

To investigate the role of the FHR proteins in vivo, we used CRISPR/Cas9 gene editing in zygotes to generate mice that lacked either the entire 664 kb FH-FHR locus (delFH-FHR) or the entire 537 kb FHR locus (delFHR) (Fig. 1*A*). For the small deletion, 200 injected zygotes led to 33 live births, of which three carried the 537 kb FHR deletion (*SI Appendix*, Fig. S1). For the large deletion, 123 injected zygotes led to 30 live births, of which four carried the 664 kb FH-FHR deletion (*SI Appendix*, Fig. S2). One delFHR founder (DGAZ-2.1c) and one delFH-FHR founder (DGCH-2.1d) were used to generate mice homozygous for the deletions. Plasma FH was reduced and absent in heterozygous and homozygous delFH-FHR mice, respectively, but normal in the delFHR strains (Fig. 1*B* and *SI Appendix*, Fig. S4*A*). Plasma FHR-C was absent in both delFH-FHR (Fig. 1*B*) and delFHR animals (*SI Appendix*, Fig. S4*B*). Consistent with absent FH levels, and comparable to what we have described in FH-deficient mice (22), the homozygous delFH-FHR mice had very low levels of plasma C3 (Fig. 1*C*) and absence of the intact C3 α-chain, indicating uncontrolled spontaneous C3 activation (*SI Appendix*, Fig. S5*A*). Plasma C3 levels in homozygous delFHR mice were comparable to wild-type and heterozygous litter mates (Fig. 1*C*), and there was no evidence of increased C3 breakdown (*SI Appendix*, Fig. S5*B*). Plasma C5 was absent on Western blot analysis in the homozygous delFH-FHR mice but detectable in delFHR animals (*SI Appendix*, Fig. S6). FH-deficient mice develop spontaneous C3G triggered by abnormal glomerular C3 deposition (22). The delFH-FHR animals developed spontaneous glomerular C3 deposition in association with properdin staining (Fig. 1*D*). We have observed mouse FHR proteins bound to glomerular C3 in FH-deficient mice (31), but as expected, this was not the case in the delFH-FHR strain (*SI Appendix*, Fig. S7). Glomerular hypercellularity and capillary wall thickening was observed in 8-mo-old delFH-FHR mice (Fig. 1*E* and *SI Appendix*, Table S2), changes comparable to aged FH-deficient mice (22). Despite this, renal function remained normal (*SI Appendix*, Table S2). The delFHR mice did not develop abnormal glomerular C3 staining, and renal histology in 8-mo-old animals was normal (Fig. 1 *D* and *E* and *SI Appendix*, Table S2). In summary, delFH-FHR mice developed spontaneous C3G, while the absence of the FHR proteins in delFHR mice did not result in either plasma C3 dysregulation or C3G.

### delFH-FHR Mice Coexpressing hFH and FHR5mut Regulate Plasma C3 in a Dose-Dependent Manner and Develop Spontaneous C3G.

**Fig. 2.**
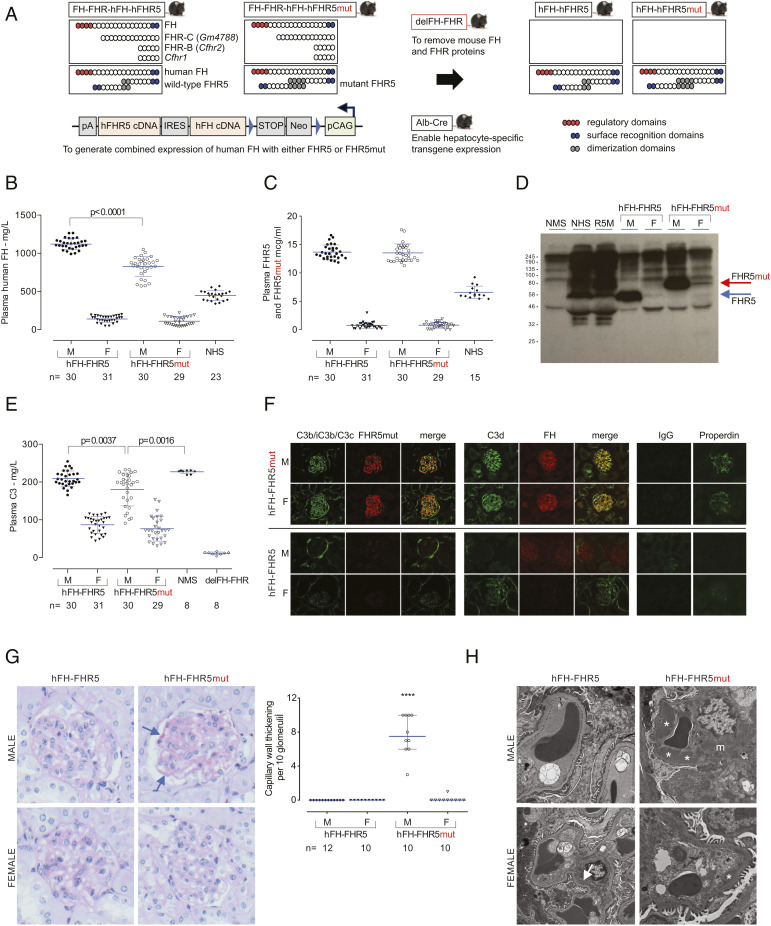
delFH-FHR mice coexpressing hFH and FHR5mut recapitulate CFHR5 nephropathy. (*A*) Schematic showing the generation of the hFH-FHR5 and hFH-FHR5mut strains. The transgenic cassette contained each cDNA in tandem, separated by an IRES, under the control of a loxP-dependent cytomegalovirus early enhancer/chicken β-actin promoter (pCAG). (*B*) Plasma hFH and (*C*) FHR5 levels. Bars denote the mean, and whiskers denote the SD. *P* values are derived from ANOVA with Bonferroni multiple comparisons test. (*D*) Western blot of FHR5 demonstrating the expression of WT and mutant FHR5 protein. Normal mouse sera (NMS), normal human sera (NHS), and FHR5 nephropathy serum (R5M) are shown. (*E*) Plasma C3 levels by ELISA. Bars denote the mean, and whiskers denote the SD. *P* values are derived from ANOVA with Bonferroni multiple comparisons test. (*F*) Representative glomerular immunostaining images of C3b/iC3b/C3c, C3d, FHR5/FHR5mut, FH, IgG, and properdin in 3-mo-old mice. (*G*) Representative glomerular images from Periodic acid–Schiff–stained renal sections from 12-mo-old mice. hFH-FHR5mut male mice show conspicuous capillary wall thickening (arrows). Scoring of capillary wall double contours in 12-mo-old mice. Bars denote the median, and whiskers denote the interquartile range. ****P < 0.0001; *P* values are derived from Kruskal–Wallis test with Dunn’s multiple comparisons test. (*H*) Representative glomerular electron microscopy images from hFH-FHR5 and hFH-FHR5mut 12-mo-old male mice. Male hFH-FHR5mut show large subendothelial electron-dense deposits (asterisks) and multiple electron-dense deposits in the mesangium (m). Smaller subendothelial deposits are seen in female hFH-FHR5mut animals. The hFH-FHR5 mice show no abnormalities apart from occasional small mesangial deposits (arrow).

To elucidate the role of FHR5mut in CFHR5 nephropathy and to investigate the role of FHR5 in glomerular C3 regulation, we generated delFH-FHR mouse strains that transgenically coexpressed hFH with either wild-type FHR5 or FHR5mut. We refer to these as hFH-FHR5 and hFH-FHR5mut strains (Fig. 2*A*). Plasma hFH and FHR5 levels in male mice were approximately twice those measured in normal human sera (Fig. 2 *B* and *C* and *SI Appendix*, Table S3). The hFH level was lower in the male hFH-FHR5mut mice compared to hFH-FHR5 animals, whereas FHR5/FHR5mut levels were similar (Fig. 2 *C* and *D*). Notably, the levels of both hFH and FHR5/FHR5mut were significantly lower in the female mice, approximating 25 and 12%, respectively, of levels in normal human sera (Fig. 2 *B*–*D* and *SI Appendix*, Fig. S8*A* and Table S3). To determine the efficacy of hFH in regulating mouse complement in vivo, we measured plasma C3 levels in the strains. Consistent with the sex difference in plasma hFH levels, plasma C3 levels were higher in the male hFH-FHR5 and hFH-FHR5mut mice compared to the respective female groups (Fig. 2*E* and *SI Appendix*, Table S3). Plasma C3 levels in the male hFH-FHR5 were equivalent to levels in normal mice and markedly elevated compared to delFH-FHR mice. The C3 levels in the hFH-FHR5mut mice were slightly lower than those in the male hFH-FHR5 and normal mice. Consistent with the differential levels of hFH, we detected more plasma C5 protein in the male compared to the female mice (*SI Appendix*, Fig. S8*B*). These data indicated that hFH was regulating mouse C3 and C5 activation in vivo in a dose-dependent manner.

Next, we analyzed the kidney. Glomerular C3 and IgG staining was normal in young male and female hFH-FHR5 mice, and there was no deposition of hFH or FHR5 (Fig. 2*F*). In contrast, in the hFH-FHR5mut animals, there was abnormal glomerular C3 staining (C3b/iC3b/C3c and C3d) together with deposition of FHR5mut, hFH, and properdin but without significant IgG staining (Fig. 2*F*). To determine if this abnormal glomerular complement resulted in renal injury, we assessed renal function and histology in 12-mo-old animals (*SI Appendix*, Table S3 and Fig. S9). There was no impairment of renal function, but there was conspicuous glomerular capillary wall thickening in all male hFH-FHR5mut animals by light microscopy (Fig. 2*G*). These changes were seen in one of the female hFH-FHR5mut mice but were absent in the hFH-FHR5 strain. Electron microscopy in 12-mo-old male hFH-FHR5mut mice showed large and conspicuous subendothelial electron-dense deposits within the glomerular capillary wall and an increase in mesangial matrix with conspicuous mesangial electron-dense deposits (Fig. 2*H*). Notably, there were no deposits in the glomerular capillary wall and only occasional small mesangial deposits in age-matched hFH-FHR5 males, expressing FHR5 levels equivalent to the FHR5mut levels in the hFH-FHR5mut male mice. In the female hFH-FHR5mut strain, subendothelial glomerular basement membrane (GBM) deposits were present but were smaller than those seen in the male hFH-FHR5mut animals. There were no deposits in the glomerular capillary wall and only occasional small mesangial deposits in age-matched hFH-FHR5 females (Fig. 2*H*). Taken together, these data indicated that FHR5mut triggered spontaneous glomerular C3 deposition in the absence of IgG, pathognomonic features of C3G.

### The FHR5mut Protein Triggers Glomerular C3 Deposition in a Dominant Manner In Vivo.

**Fig. 3.**
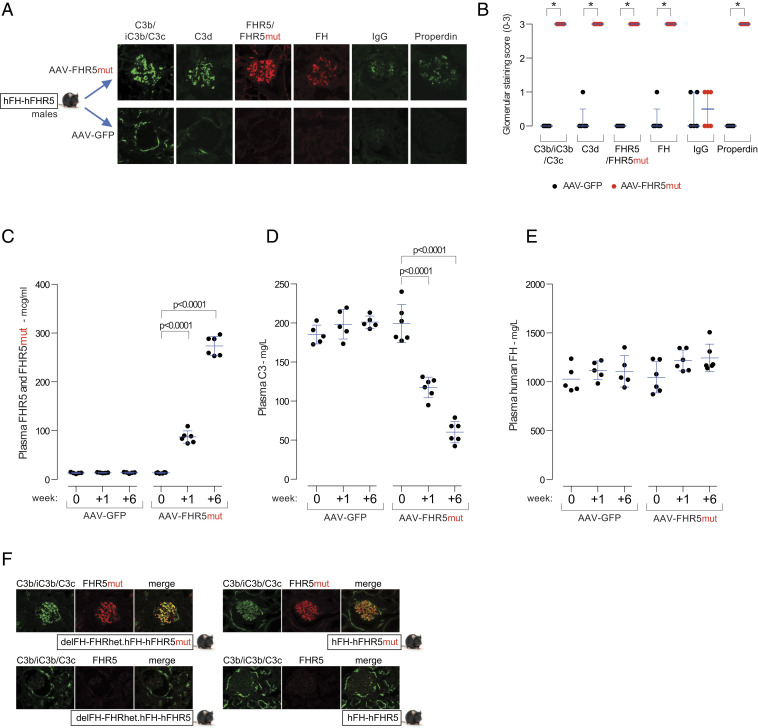
FHR5mut triggers glomerular C3 deposition in hFH-FHR5 mice. (*A*) Representative glomerular images and (*B*) staining scores for C3b/iC3b/C3c, C3d, FHR5/FHR5mut, FH, IgG, and properdin in male hFH-FHR5 mice treated with either AAV-GFP (*n* = 5, black dots) or AAV-FHR5mut (*n* = 6, red dots). Bars denote the median, and whiskers denote the interquartile range. *P = 0.0022; values are derived from Mann–Whitney *U* test. Plasma FHR5/FHR5mut (*C*), C3 (*D*), and FH (*E*) levels after administration of either AAV-FHR5mut or AAV-GFP to hFH-FHR5 male mice. Bars denote the mean, and whiskers denote the SD. *P* values are derived from two-way ANOVA with Bonferroni multiple comparisons test. (*F*) Representative glomerular images of C3b/iC3b/C3c, C3d, and FHR5mut in male hFH-FHR5mut mice expressing one copy of the transgene on the background of either heterozygous (delFH-FHRhet.hFH-FHR5mut) or homozygous (hFH-FHR5mut) deficiency of the mouse FH-FHR locus. Controls are male delFH-FHRhet.hFH-FHR5 and hFH-FHR5 animals.

CFHR5 nephropathy is an autosomal dominant condition, and, to date, all reported cases have been heterozygous for the *CFHR5* mutation. We next examined if FHR5mut could trigger glomerular C3 deposition in male hFH-FHR5 mice. We expressed FHR5mut in hFH-FHR5 male mice using a hepatotropic AAV vector (Fig. 3). Glomerular complement staining 6 wk after injection of the AAV-FHR5mut demonstrated abnormal glomerular C3 staining together with staining for FH and FHR5/FHR5mut proteins but minimal IgG staining (Fig. 3 *A* and *B*). Compared to preinjection levels, plasma FHR5 (consisting of transgene-derived FHR5 and AAV-derived FHR5mut) increased 6-fold at week one and 20-fold by week six (Fig. 3*C*). Plasma hFH levels did not change, but there was a progressive fall in plasma C3 levels in the AAV-FHR5mut group (Fig. 3 *D* and *E*). Further evidence for a dominant gain-of-function role of FHR5mut in C3G pathogenesis derived from observations in male mice heterozygous for the FH-FHR locus and expressing one copy of the hFH-FHR5mut transgene (delFH-FHRhet.hFH-FHR5mut). Despite the presence of mouse FH (derived from the single intact mouse FH-FHR allele) and hFH (derived from the single hFH-FHR5mut transgene), there was spontaneous abnormal glomerular C3 staining in association with FHR5mut (Fig. 3*F* and *SI Appendix*, Fig. S10). This developed despite normal plasma C3 levels (*SI Appendix*, Fig. S10). Glomerular C3 and FHR5 staining was normal in male delFH-FHRhet.hFH-FHR5 and hFH-FHR5 animals. Taken together, these data show that FHR5mut exerts a dominant gain-of-function effect in vivo.

### HDM-FH Ameliorates Glomerular C3 Deposition Associated with the FHR5mut Protein.

**Fig. 4.**
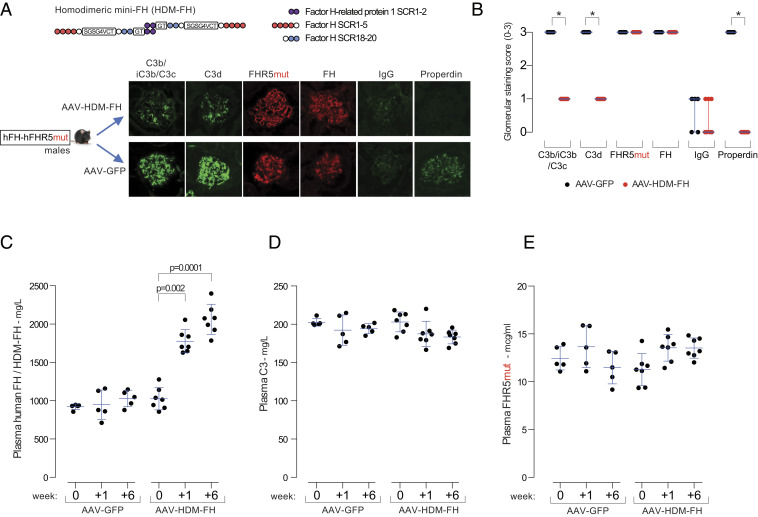
HDM-FH ameliorates glomerular C3 in hFH-FHR5mut mice. (*A*) Representative glomerular images and (*B*) staining scores for C3b/iC3b/C3c, C3d, FHR5mut, FH, IgG, and properdin in male hFH-FHR5mut mice treated with either AAV-GFP (*n* = 5, black dots) or AAV-HDM-FH (*n* = 7). Bars denote the median, and whiskers denote the interquartile range. *P = 0.0013, derived from Mann–Whitney *U* test. Plasma hFH/HDM-FH (*C*), C3 (*D*), and FHR5mut (*E*) levels after administration of either AAV-GFP or AAV-HDM-FH to hFH-FHR5mut male mice. Bars denote the mean, and whiskers denote the SD. *P* values are derived from two-way ANOVA with Bonferroni multiple comparisons test.

The presence of FHR5mut is associated with abnormal renal C3 deposition despite circulating hFH, suggesting that it promotes glomerular C3 activation by impairing the ability of FH to negatively regulate C3 activation in situ. To test this, we investigated if HDM-FH, a molecule with enhanced affinity for surface C3b compared to FH (30), could alter glomerular C3. We administered AAV-HDM-FH to male hFH-FHR5mut mice (Fig. 4). At 6 wk after injection, there was a significant reduction in glomerular C3b/iC3b/C3c, C3d, and properdin staining in the AAV-HDM-FH–treated mice (Fig. 4 *A* and *B*). Notably, we could not detect a change in either glomerular FHR5 or FH staining. Using a hFH enzyme linked immunosorbent assay (ELISA), which detects both transgene-derived FH and AAV-derived HDM-FH, levels increased at week one and week six in the AAV-HDM-FH–treated mice, indicating successful expression of the HDM-FH molecule (Fig. 4*C*). Plasma FHR5 and C3 levels did not change (Fig. 4 *D* and *E*). These data demonstrated that HDM-FH ameliorated FHR5mut-induced glomerular C3 deposition.

## Discussion

We modeled CFHR5 nephropathy in vivo by generating mouse strains that expressed hFH with either the normal FHR5 protein or FHR5mut, the abnormal FHR5 protein associated with CFHR5 nephropathy. Remarkably, the male hFH-FHR5mut phenotype recapitulated the key features of CHFR5 nephropathy: normal FH function, intact plasma C3 regulation, abnormal glomerular C3 deposition in the absence of IgG, and the development of electron-dense deposits within the GBM and mesangial matrix.

Evidence for a dominant gain-of-function effect of the FHR5mut protein was derived from our observation that abnormal glomerular C3 deposition in association with FHR5mut was also present in mice heterozygous for the delFH-FHR allele but expressing one copy of the hFH-FHR5mut transgene. Both hFH and mouse FH (derived from the intact mouse FH-FHR locus) were unable to prevent FHR5mut-mediated glomerular C3 deposition. Moreover, AAV-mediated expression of the FHR5mut protein in male hFH-FHR5 animals triggered glomerular C3 deposition, demonstrating a direct role for FHR5mut in this process. In this experiment, FHR5mut levels were very high in comparison to human levels and those in our hFH-FHR5mut animals, and we detected a progressive fall in plasma C3. We do not know the cause for this. Plasma C3 levels are typically normal in CFHR5 nephropathy (3, 10). However, in patients with C3G due to a CFHR2-CFHR5 hybrid gene, low plasma C3 levels have been reported, and the FHR2-FHR5 mutant protein was shown to stabilize the C3 convertase (2).

The prevailing hypothesis to explain the development of excessive glomerular C3 deposition in FHR5 nephropathy is that under normal circumstances, there is activation of C3 within glomeruli. FH is critical for inhibiting this, particularly on noncell surfaces (i.e., the GBM and mesangial matrix). These structures, unlike glomerular cells, lack additional complement regulators. If FH function is impaired, these structures become complement-activating surfaces and abnormal C3 accumulation occurs. We have previously shown that FH-deficient mice develop spontaneous C3 deposition along the GBM (22). In the hFH-FHR5mut strain, the FHR5mut protein, through increased avidity for C3 ligands, interacts with surface C3 within glomeruli more efficiently than hFH and promotes complement activation in situ. Consistent with this hypothesis, the interaction between FHR5 and surface C3 enabled C3 convertase assembly and further in situ C3 activation in vitro (17, 32). Furthermore, both FHR5 and FHR5mut have increased avidity for C3b compared to FH, and in vitro FHR5 can out-compete FH for surface C3b binding and promote complement activation (FH deregulation) (2, 16). In this study, we show in vivo that administration of HDM-FH to the male hFH-FHR5mut mice reduced glomerular C3 staining. HDM-FH is a mini-FH molecule (28) containing the oligomerization domains of FHR1. Critically, HDM-FH has been shown to be >10-fold more effective than hFH in inhibiting surface complement activation (30). Furthermore, it was more efficient in reducing glomerular C3 in FH-deficient animals than either FH or mini-FH (30). Similarly, the C3 staining intensity was reduced in the HDM-FH–treated hFH-FHR5mut mice because of the higher affinity of HDM-FH for glomerular C3. Notably, the intensity of glomerular FHR5mut staining did not change in this experiment. This likely reflects the FHR5mut-C3d interaction that is stable and uninfluenced by hFH or HDM-FH. Glomerular FHR5, in association with C3d, is a frequent finding in C3G and is considered a marker of previous glomerular complement activation (33).

The role of the murine FHR proteins in complement-mediated renal disease models has not been studied to date. Mouse FHR proteins have been shown to both deregulate FH (21) and promote complement activation (34) and therefore functionally recapitulate the actions reported for the human FHR proteins. Importantly, our data showed that the delFHR animals have normal glomerular and plasma C3 regulation. A reduction in FHR activity is functionally analogous to the situation present in individuals with the ∆*CFHR3-1* allele and, in this respect, the delFHR strain will be an informative model to investigate if mouse FHR proteins modulate glomerular complement in renal disease models. The delFH-FHR strain recapitulated the phenotype we reported in FH-deficient mice. By comparing the two strains, we demonstrated the presence of FHR proteins in association with glomerular complement in the FH-deficient strain. However, the relative proportions of the different mouse FHR proteins present in the FH-deficient mice remain unknown. It is likely that the contribution of FHR-B and FHR-C will differ in vivo since recent in vitro data has shown that, while FHR-B mediated FH deregulation, FHR-C did not (21). There was no obvious difference in the phenotypes between the delFH-FHR and FH-deficient mice, indicating that in the setting of extreme complement dysregulation (i.e., complete FH deficiency), the actions of the FHR proteins become irrelevant.

A potential limitation to our hFH-FHR5mut model was that we did not express the other human FHR proteins in the mice. FHR1, FHR2, and FHR5 exist as obligate homodimers through shared dimerization domains (7, 16), and FHR1-FHR2 heterodimers have been detected in human plasma (35). Mutations in FHR1 and a hybrid FHR3-1 gene are also associated with C3G (4, 7) but, to date, have not been modeled in vivo. FHR5mut and mutations in FHR1 (7) may result in the formation of large multimers. We do not know the relevance of this or the influence of surface glycan binding (20) in our model. A technical limitation in the glomerular complement phenotyping of the hFH-FHR5 and hFH-FHR5mut strains was that we were unable to distinguish the FHR5 and FHR5mut proteins using immunostaining. Therefore, in the experiment in which we expressed AAV-FHR5mut in hFH-FHR5 animals, it was not possible to determine the relative amounts of FHR5/FHR5mut in glomeruli.

There was a marked sex difference in the expression of the transgenes. Female mice in both the hFH-FHR5 and hFH-FHR5mut strains expressed the proteins at significantly lower levels than the respective male mice (for example, an eightfold difference in plasma FH levels between male and female hFH-FHR5 mice). For the purposes of modeling CFHR5 nephropathy in vivo, this was not problematic, as male mice had adequate levels of the proteins in plasma. Furthermore, the range of protein differences between the sexes was, serendipitously, highly informative in demonstrating the dominant gain-of-function role of the FHR5mut protein in glomerular C3 deposition. While there is a striking sex difference in the incidence of renal failure in CFHR5 nephropathy (much higher in males), the reasons are not related to large intersex differences in circulating FH and FHR5 levels. Although progression to renal impairment is associated with episodes of synpharyngitic macroscopic hematuria, suggesting a role for infection (10), the causes for the male predominance in chronic renal failure in CFHR5 nephropathy is not known.

We have not investigated the cause of the sexually dimorphic expression of the transgenes in our strains, but this phenomenon is well reported in general, and causes include position-effect variegation and epigenetic modification (36).

In summary, these data demonstrate that in the presence of FHR5mut, the extracellular surfaces within the glomeruli (i.e., the mesangial matrix and GBM) can become activating surfaces for C3 deposition, despite the presence of FH, and that the abnormal C3 accumulation in this setting could be ameliorated by a FH-derived regulator engineered to have enhanced surface complement regulatory activity. These findings provide a rationale to investigate this approach in the treatment of C3G and further illustrate the important role of the balance between FHR and FH activity in surface C3 activation.

## Materials and Methods

### Generation of delFH-FHR and delFHR Strains.

CRISPR/Cas9 target sites were identified using crispr.mit.edu/as (37), and the guide RNAs (gRNAs) were selected following the guidelines from ref. 38, avoiding C and T upstream of the protospacer adjacent motif (PAM), G downstream of the PAM, and T within the PAM whenever possible. Pairs of gRNAs were designed for the 5′ and 3′ end of the locus to be deleted (*SI Appendix*, Table S1). These were typically located within 50 to 200 bp of each other and positioned on opposite strands. Single-strand oligonucleotides (ssODNs), designed to bridge the deletions, were 120 bp in length and positioned directly adjacent to the most external gRNA site (*SI Appendix*, Table S1). These bridging oligonucleotides were intended to act as a repair template for homology-directed repair of the deletion, coordinating the deletion to a specific genomic location at the single nucleotide level. The gRNA oligonucleotides were synthesized and the two strands annealed and cloned into a vector containing the gRNA backbone and a T7 promoter for RNA production using BsaI. For Cas9 mRNA production, the vector from ref. 39 was modified to contain the T7 promoter. The integrity of all plasmids was confirmed by DNA sequencing. For Cas9 RNA production, the T7/Cas9 plasmid was linearized with EcoRI and for gRNA production with DraI. The plasmids were cleaned with a PCR purification kit (Qiagen) and in vitro transcribed using mMessage mMachine T7 Ultra kit and MEGAshortscript T7 kits (Life Technologies), respectively. Both Cas9 mRNA and gRNA were purified using the MEGAclear kit (Life Technologies) and eluted in ribonuclease (RNase)-free water. The quality of the RNA was analyzed using Agilent RNA 6000 Nano kit (Agilent Technologies, 2100 Bioanalyzer) and Qubit RNA high sensitivity assay kit (Life Technologies). The ssODNs were synthesized by Integrated DNA Technologies and dissolved in RNase-free water to a concentration of 1,000 ng/uL. Zygote injections were performed using standard procedures described in *SI Appendix*, *Supplemental Methods*. Genotyping was performed on lysates of genomic DNA isolated from ear clips using the KAPA Mouse Genotyping kit containing KAPA 2G HotStart DNA polymerase. Genotyping primers are described in *SI Appendix*, Table S1. Probes used for fluorescent in situ hybridization (FISH) are listed in *SI Appendix*, Fig. S3, and the Fiber-FISH procedure is described in *SI Appendix*, *Supplemental Methods*.

### Complement Phenotyping.

Mouse plasma was collected from mice in the presence of EDTA by cardiac puncture. For Western blotting, samples were separated using sodium dodecyl sulphate-polyacrylamide gel electrophoresis (SDS-PAGE) under reducing conditions for C3 and nonreducing conditions for mouse C5, hFH, mouse FH, FHR-C (Gm4788), and FHR5. For detection antibodies, goat anti-mouse C3 (MP Biomedicals, 55444), rat anti-mouse FH (R&D Systems, MAB4999 Clone #511419), sheep anti-mouse FH (to detect FHR-C [Gm4788]; R&D Systems AF4999), goat anti-hFH (Quidel A312), rabbit anti-human FHR5 (Abnova H00081494-D01P), goat anti-human C5 (Quidel, A306), and 402H/Y-specific anti-hFH (provided by Prof Paul Morgan, University of Cardiff) were used. For secondary antibodies, goat anti-rat horseradish peroxidase (HRP) (Biolegend, 405405), mouse anti-goat/sheep IgG-HRP (Sigma, A9452), and swine anti-rabbit IgG-HRP (Dako, P0217) were used. Blots were visualized using enhanced chemiluminescence (ECL) Western Blotting Substrate (Pierce, Thermo). For the measurement of mouse C3, mouse FH, hFH, and FHR5 levels by ELISA, mouse C3 was detected using goat anti-mouse C3 antibody (MP Biomedicals, 55463) in combination with an HRP-conjugated goat anti-mouse C3 polyclonal antibody (MP Biomedicals, 55557). The results were quantified using a standard curve generated from pooled mouse sera containing a known quantity of C3. Mouse FH levels were detected using a polyclonal sheep anti-hFH antibody (ABIN 113017) and a biotinylated goat anti-hFH antibody (Quidel, A312). FH levels in the samples were plotted as optical density. For hFH levels, ELISA plates were coated with 2 µg/mL anti-FH monoclonal OX24 antibody (Thermo Scientific MA1-70057) in 0.2 M carbonate buffer. After washing and blocking with PBS–1% bovine serum albumin (BSA), mouse plasma (1: 20,000) was added in PBS–0.1% Tween 20–1% BSA. After incubation and washing, plates were incubated with a polyclonal sheep anti-hFH antibody (Abcam, ab8842) for 1 h (1:20,000). After washing, plates were incubated with a Donkey anti-sheep, HRP-conjugated antibody (Jackson ImmunoResearch, 713-035-003) for 1 h (1:20,000). After washing, enzymatic activity was developed using 3,3’,5,5’-tetramethylbenzinidine (TMB) substrate. The results were quantified using a standard curve generated using purified hFH (Complement Technologies). FHR5 levels were measured by ELISA using rabbit monoclonal anti-FHR5 antibody (Abnova, H00081494-D01P) as the capture antibody. A mouse monoclonal anti-FHR5 antibody (Abnova, H00081494-B01P) was used as the detecting antibody. The results were quantified using a standard curve generated using purified human FHR5 (R&D).

### Renal Phenotyping.

Hematuria and proteinuria were assessed using Hema-Combistix (Siemens); plasma urea was measured using an enzymatic ultraviolet method according to the manufacturer’s instructions (R-Biopharm). Mouse albumin levels were measured using a Mouse Albumin ELISA kit according to the manufacturer’s instructions (Cambridge Bioscience). For glomerular histological analysis, kidneys were fixed in Bouin’s solution (Sigma) as previously described (25), and Periodic acid–Schiff stained sections were examined by light microscopy. Sections were scored semiquantitatively for glomerular cellularity (0 to 4), segmental sclerosis (0 to 1), and mesangial expansion (0 to 1). Capillary wall thickening was assessed as the number of glomeruli showing conspicuous capillary wall thickening in 10 consecutive glomeruli examined. Immunofluorescence (IF) staining was performed on 5 µm cryosections mounted using Vectashield medium with DAPI (Vector Laboratories). IF antibodies were as follows: C3b/iC3b/C3c, fluorescein isothiocyanate (FITC)-conjugated goat anti-mouse C3 (MP Biomedicals, 855500); C3d, biotinylated goat anti-mouse C3d (R&D system, BAF2655); mouse FH and mouse FHR proteins, polyclonal goat anti-hFH (Quidel), A312; hFH, polyclonal goat anti-hFH (Quidel, A312); FHR5/FHR5mut, rabbit monoclonal anti-human CFHR5 antibody (Abnova, H00081494-D01P); mouse properdin, FITC-conjugated goat anti-human properdin (Caltag Madsystems, GAHu/PPD/FITC); and mouse IgG, FITC-conjugated polyclonal goat anti-mouse IgG Fc γ-chain–specific antibody (Sigma-Aldrich, F5387). Glomerular macrophages were identified using FITC-conjugated rat anti-mouse CD68 (GeneTex; FA-11 clone; GTX43518). IF analysis was performed using a Leica DM4B optical microscope coupled with Leica DFC700T digital camera (Leica Microsystems). A total of 10 glomeruli were examined per section. Categorical IF scoring was performed using a staining intensity scale (0: normal/absent, 1: mild, 2: moderate, and 3: strong). Electron microscopy was performed on mouse tissue fixed using glutaraldehyde buffer as previously described (22).

### AAV Experiments.

We utilized the hepatotropic serotype eight capsid to enable robust transgene expression in the adult mouse liver following a single intraperitoneal injection of AAV vector (40). The complementary DNA (cDNA) for FHR5, FHR5mut, and HDM-FH each containing a Kozak consensus sequence was inserted into an AAV vector construct (41) with a modified promoter sequentially containing one copy of the ApoE enhancer, a human 1-α-antitrypsin promoter, and SV40 intron (42). AAV vector constructs were packaged by the Vector and Genome Engineering Facility (Children’s Medical Research Institute) as previously described (43), except that transfections were performed using PEI. AAV vectors were purified (44) and titred by qPCR (40). Animals were intraperitoneally injected with 1 × 10^12^ vector genomes of the relevant AAV vector. The following vectors used were: AAV-FHR5mut, AAV-GFP, and AAV-HDM-FH. The generation of HDM-FH has been described previously (30). It contains the oligodimerization domains of FHR1 (SCR1-2) linked to mini-FH. Mini-FH consists of the complement regulatory domains of hFH (SCR1-5) linked to the surface recognition domains (SCR18-20).

### Generation of hFH-FHR5 or hFH-FHR5mut Mice.

The humanized strains were generated by genOway using the Rosa26 Quick Knockin targeting vector (www.genoway.com). The cDNA cassettes containing hFH with either FHR5 (hFH-IRES-hFHR5 cDNA) or FHR5mut (hFH-IRES-hFHR5mut cDNA) downstream of a CAG promoter were inserted into the Rosa26 locus in C57BL/6 embryonic stem (ES) cells. The genOway Knockin vector contained a lox P-flanked STOP cassette inserted between the CAG promoter and the coding sequence of the transgene to ensure expression only in the presence of Cre recombinase. The vector is isogenic with C57BL/6 ES cells. To generate heterozygous inducible hFH-hFHR5/hFH-FHR5mut mice, chimeras were crossed with C57BL/6N wild-type mice. The hFH-FHR5 and hFH-FHR5mut strains were generated by intercrossing with Alb-Cre (to generate hepatocyte-specific expression of the proteins) and with the delFH-FHR mice to remove the mouse FH and FHR proteins. Alb-Cre mice were purchased from the Jackson Laboratories (B6.Cg-Tg[Alb-cre]21Mgn/J; strain #003574). Genotyping was performed by PCR on digested tissue. Genomic PCR was performed using Longamp Taq DNA Polymerase with 10 ng genomic DNA according to the manufacturer’s protocol. The Cre allele PCR was performed using Megamix blue DNA polymerase (Clent Life Sciences) with 10 ng genomic DNA according to the manufacturer’s protocol. Primers to detect the Alb-Cre allele were 5′-CGT​ACT​GAC​GGT​GGG​AGA​AT-3′ and 5′-CCC​GGC​AAA​ACA​GGT‐AGT​TA-3′, which generated a 178 bp amplicon. Primers to detect the hFH-FHR5/hFH-FHR5mut allele were 5′-GTT​TTG​GAG​GCA​GGA​AGC​ACT​TGC-3′, 5′-CAA​TGC​TCT​GTC​TAG​GGG​TTG​GAT​AAG​C-3′, and 5′-GCA​GTG​AGA​AGA​GTA‐CCA​CCA​TGA​GTC​C-3′, generating either a 700 bp amplicon (wild-type allele) or 270 bp amplicon (transgene allele). Primers to detect the delFH-FHR allele were 5′-GTA​AAG​GTC​CTC​CTC​CAA​GAG-3′ and 5′-GGT​ATA​AAC​AAC​CTT​TGC‐ACC-3′, generating a 600 bp amplicon (wild-type allele), or 5′-TCC​TGA​AGG‐CTG​GAA​CAA​GT-3′ and 5′-TAA​ACA​AGG​CAG​GAG​GGA​TG-3′, generating a 324 bp amplicon (delFH-FHR allele).

### Mice.

All mice were housed in specific, pathogen-free conditions, and procedures were performed according to institutional guidelines and approved by the UK Home Office.

### Statistical Analysis.

Statistical analysis was performed using GraphPad Prism (version 8.0; GraphPad). ANOVA with Bonferroni multiple comparisons test was used when comparing parametric data from multiple groups. Repeated-measures two-way ANOVA with Bonferroni multiple comparisons test (factors were time and treatment) was used for time course analyses. Kruskal–Wallis with Dunn’s multiple comparisons test was used when comparing nonparametric data from multiple groups and Mann–Whitney *U* test for two groups.

## Supplementary Material

Supplementary File

## Data Availability

All study data are included in the article and/or *SI Appendix*.

## References

[1] M. C. Pickering, H. T. Cook, Translational mini-review series on complement factor H: Renal diseases associated with complement factor H: Novel insights from humans and animals. Clin. Exp. Immunol. 151, 210–230 (2008). 18190458 10.1111/j.1365-2249.2007.03574.x PMC2276951

[2] Q. Chen., Complement factor H-related hybrid protein deregulates complement in dense deposit disease. J. Clin. Invest. 124, 145–155 (2014). 24334459 10.1172/JCI71866 PMC3871254

[3] D. P. Gale., Identification of a mutation in complement factor H-related protein 5 in patients of Cypriot origin with glomerulonephritis. Lancet 376, 794–801 (2010). 20800271 10.1016/S0140-6736(10)60670-8 PMC2935536

[4] T. H. Malik., A hybrid CFHR3-1 gene causes familial C3 glomerulopathy. J. Am. Soc. Nephrol. 23, 1155–1160 (2012). 22626820 10.1681/ASN.2012020166 PMC3380655

[5] N. Medjeral-Thomas., A novel CFHR5 fusion protein causes C3 glomerulopathy in a family without Cypriot ancestry. Kidney Int. 85, 933–937 (2014). 24067434 10.1038/ki.2013.348 PMC3789233

[6] S. K. Togarsimalemath., A novel CFHR1-CFHR5 hybrid leads to a familial dominant C3 glomerulopathy. Kidney Int. 92, 876–887 (2017). 28729035 10.1016/j.kint.2017.04.025

[7] A. Tortajada., C3 glomerulopathy-associated CFHR1 mutation alters FHR oligomerization and complement regulation. J. Clin. Invest. 123, 2434–2446 (2013). 23728178 10.1172/JCI68280 PMC3668852

[8] X. Xiao., Familial C3 glomerulonephritis caused by a novel CFHR5-CFHR2 fusion gene. Mol. Immunol. 77, 89–96 (2016). 27490940 10.1016/j.molimm.2016.07.007

[9] H. T. Cook, M. C. Pickering, Histopathology of MPGN and C3 glomerulopathies. Nat. Rev. Nephrol. 11, 14–22 (2015). 25447133 10.1038/nrneph.2014.217

[10] Y. Athanasiou., Familial C3 glomerulopathy associated with CFHR5 mutations: Clinical characteristics of 91 patients in 16 pedigrees. Clin. J. Am. Soc. Nephrol. 6, 1436–1446 (2011). 21566112 10.2215/CJN.09541010 PMC3109942

[11] A. G. Gharavi., Genome-wide association study identifies susceptibility loci for IgA nephropathy. Nat. Genet. 43, 321–327 (2011). 21399633 10.1038/ng.787 PMC3412515

[12] N. R. Medjeral-Thomas., Circulating complement factor H-related proteins 1 and 5 correlate with disease activity in IgA nephropathy. Kidney Int. 92, 942–952 (2017). 28673452 10.1016/j.kint.2017.03.043 PMC5611987

[13] A. Tortajada., Elevated factor H-related protein 1 and factor H pathogenic variants decrease complement regulation in IgA nephropathy. Kidney Int. 92, 953–963 (2017). 28637589 10.1016/j.kint.2017.03.041

[14] N. R. Medjeral-Thomas., Progressive IgA nephropathy is associated with low circulating mannan-binding lectin-associated serine protease-3 (MASP-3) and increased glomerular factor H-related protein-5 (FHR5) deposition. Kidney Int. Rep. 3, 426–438 (2017). 29725647 10.1016/j.ekir.2017.11.015 PMC5932138

[15] L. Zhu., Circulating complement factor H-related protein 5 levels contribute to development and progression of IgA nephropathy. Kidney Int. 94, 150–158 (2018). 29759419 10.1016/j.kint.2018.02.023

[16] E. Goicoechea de Jorge., Dimerization of complement factor H-related proteins modulates complement activation in vivo. Proc. Natl. Acad. Sci. U.S.A. 110, 4685–4690 (2013). 23487775 10.1073/pnas.1219260110 PMC3606973

[17] A. I. Csincsi., Factor H-related protein 5 interacts with pentraxin 3 and the extracellular matrix and modulates complement activation. J. Immunol. 194, 4963–4973 (2015). 25855355 10.4049/jimmunol.1403121 PMC4416742

[18] A. I. Csincsi., FHR-1 binds to C-reactive protein and enhances rather than inhibits complement activation. J. Immunol. 199, 292–303 (2017). 28533443 10.4049/jimmunol.1600483

[19] M. Hebecker, M. Józsi, Factor H-related protein 4 activates complement by serving as a platform for the assembly of alternative pathway C3 convertase via its interaction with C3b protein. J. Biol. Chem. 287, 19528–19536 (2012). 22518841 10.1074/jbc.M112.364471 PMC3365989

[20] M. Józsi, A. Tortajada, B. Uzonyi, E. Goicoechea de Jorge, S. Rodríguez de Córdoba, Factor H-related proteins determine complement-activating surfaces. Trends Immunol. 36, 374–384 (2015). 25979655 10.1016/j.it.2015.04.008

[21] A. H. Antonioli., Modulation of the alternative pathway of complement by murine factor H-related proteins. J. Immunol. 200, 316–326 (2018). 29187587 10.4049/jimmunol.1602017 PMC5736413

[22] M. C. Pickering., Uncontrolled C3 activation causes membranoproliferative glomerulonephritis in mice deficient in complement factor H. Nat. Genet. 31, 424–428 (2002). 12091909 10.1038/ng912

[23] M. C. Pickering., Spontaneous hemolytic uremic syndrome triggered by complement factor H lacking surface recognition domains. J. Exp. Med. 204, 1249–1256 (2007). 17517971 10.1084/jem.20070301 PMC2118613

[24] K. L. Rose., Factor I is required for the development of membranoproliferative glomerulonephritis in factor H-deficient mice. J. Clin. Invest. 118, 608–618 (2008). 18202746 10.1172/JCI32525 PMC2200299

[25] M. M. Ruseva., Loss of properdin exacerbates C3 glomerulopathy resulting from factor H deficiency. J. Am. Soc. Nephrol. 24, 43–52 (2013). 23184055 10.1681/ASN.2012060571 PMC3537217

[26] E. G. de Jorge., The development of atypical hemolytic uremic syndrome depends on complement C5. J. Am. Soc. Nephrol. 22, 137–145 (2011). 21148255 10.1681/ASN.2010050451 PMC3014042

[27] F. Fakhouri., Treatment with human complement factor H rapidly reverses renal complement deposition in factor H-deficient mice. Kidney Int. 78, 279–286 (2010). 20445496 10.1038/ki.2010.132 PMC2906702

[28] E. M. Nichols., An extended mini-complement factor H molecule ameliorates experimental C3 glomerulopathy. Kidney Int. 88, 1314–1322 (2015). 26221753 10.1038/ki.2015.233 PMC4650264

[29] M. C. Pickering., Prevention of C5 activation ameliorates spontaneous and experimental glomerulonephritis in factor H-deficient mice. Proc. Natl. Acad. Sci. U.S.A. 103, 9649–9654 (2006). 16769899 10.1073/pnas.0601094103 PMC1476693

[30] Y. Yang., An engineered complement factor H construct for treatment of C3 glomerulopathy. J. Am. Soc. Nephrol. 29, 1649–1661 (2018). 29588430 10.1681/ASN.2017091006 PMC6054357

[31] D. Paixão-Cavalcante, S. Hanson, M. Botto, H. T. Cook, M. C. Pickering, Factor H facilitates the clearance of GBM bound iC3b by controlling C3 activation in fluid phase. Mol. Immunol. 46, 1942–1950 (2009). 19411110 10.1016/j.molimm.2009.03.030 PMC2697322

[32] É. Kárpáti., Interaction of the factor H family proteins FHR-1 and FHR-5 with DNA and dead cells: Implications for the regulation of complement activation and opsonization. Front. Immunol. 11, 1297 (2020). 32765490 10.3389/fimmu.2020.01297 PMC7378360

[33] N. R. Medjeral-Thomas., Glomerular complement factor H-related protein 5 (FHR5) is highly prevalent in C3 glomerulopathy and associated with renal impairment. Kidney Int. Rep. 4, 1387–1400 (2019). 31701048 10.1016/j.ekir.2019.06.008 PMC6829196

[34] M. Cserhalmi., The murine factor H-related protein FHR-B promotes complement activation. Front. Immunol. 8, 1145 (2017). 28974948 10.3389/fimmu.2017.01145 PMC5610720

[35] A. E. van Beek., Factor H-related (FHR)-1 and FHR-2 form homo- and heterodimers, while FHR-5 circulates only as homodimer in human plasma. Front. Immunol. 8, 1328 (2017). 29093712 10.3389/fimmu.2017.01328 PMC5651247

[36] C. W. Lai., Sexually dimorphic expression of eGFP transgene in the Akr1A1 locus of mouse liver regulated by sex hormone-related epigenetic remodeling. Sci. Rep. 6, 24023 (2016). 27087367 10.1038/srep24023 PMC4834580

[37] F. A. Ran., Genome engineering using the CRISPR-Cas9 system. Nat. Protoc. 8, 2281–2308 (2013). 24157548 10.1038/nprot.2013.143 PMC3969860

[38] J. G. Doench., Rational design of highly active sgRNAs for CRISPR-Cas9-mediated gene inactivation. Nat. Biotechnol. 32, 1262–1267 (2014). 25184501 10.1038/nbt.3026 PMC4262738

[39] L. Cong., Multiplex genome engineering using CRISPR/Cas systems. Science 339, 819–823 (2013). 23287718 10.1126/science.1231143 PMC3795411

[40] A. P. Dane, S. J. Wowro, S. C. Cunningham, I. E. Alexander, Comparison of gene transfer to the murine liver following intraperitoneal and intraportal delivery of hepatotropic AAV pseudo-serotypes. Gene Ther. 20, 460–464 (2013). 22895507 10.1038/gt.2012.67

[41] S. C. Cunningham, A. P. Dane, A. Spinoulas, I. E. Alexander, Gene delivery to the juvenile mouse liver using AAV2/8 vectors. Mol. Ther. 16, 1081–1088 (2008). 10.1038/mt.2008.72 28178471

[42] A. C. Nathwani., Self-complementary adeno-associated virus vectors containing a novel liver-specific human factor IX expression cassette enable highly efficient transduction of murine and nonhuman primate liver. Blood 107, 2653–2661 (2006). 16322469 10.1182/blood-2005-10-4035 PMC1895379

[43] X. Xiao, J. Li, R. J. Samulski, Production of high-titer recombinant adeno-associated virus vectors in the absence of helper adenovirus. J. Virol. 72, 2224–2232 (1998). 9499080 10.1128/jvi.72.3.2224-2232.1998 PMC109519

[44] E. Ayuso., High AAV vector purity results in serotype- and tissue-independent enhancement of transduction efficiency. Gene Ther. 17, 503–510 (2010). 19956269 10.1038/gt.2009.157

