# Supplementary material for: Gain-of-function factor H–related 5 protein impairs glomerular complement regulation resulting in kidney damage
Source: Proc Natl Acad Sci U S A. 2021 Mar 22;118(13):e2022722118. doi: 10.1073/pnas.2022722118 (PMC8020653; doi:10.1073/pnas.2022722118)
Supplement: Supplementary File [file pnas.2022722118.sapp.pdf]

SUPPLEMENTAL DATA

Mutant factor H-related 5 protein impairs glomerular complement regulation resulting in kidney damage

AUTHORS:

Talat H Malik[1*], Daniel P Gitterman[2*], Deborah P Lavin[1*], Hannah J Lomax-Browne[1], E. Christina Hiemeyer[1], Linda B Moran[3], Katharina Boroviak[2], H. Terence Cook[1], Alyssa C. Gilmore[1], Mawj Mandwie[4], Amina Ahmad[4], Ian E Alexander[4, 5], Grant J Logan[4], Kevin J Marchbank[6], Allan Bradley[2], Matthew C. Pickering[1].

[1]Centre for Inflammatory Disease, Imperial College London, UK;

[2]Wellcome Trust Sanger Institute, Cambridge, UK;

[3]North West London Pathology, Imperial College Healthcare NHS Trust, London, UK;

[4]Gene Therapy Research Unit, Children's Medical Research Institute and Sydney Children's Hospitals Network, The University of Sydney, Westmead, NSW 2145, Australia;

[5]Discipline of Child and Adolescent Health, The University of Sydney, Sydney Medical School, Faculty of Medicine and Health, Westmead, NSW 2145, Australia;

[6]Translational and Clinical Institute, Newcastle University and National Renal Complement Therapeutics Centre, Royal Victoria Infirmary, Newcastle upon Tyne, UK;

*These authors contributed equally to this work

CONTENTS:

Supplemental Figure 1. Generation of the delFHR allele.

Supplemental Figure 2. Generation of the delFH-FHR allele.

Supplemental Figure 3. Fibre FISH probes across the FH-FHR region.

Supplemental Figure 4. Plasma FH and FHR-C in delFHR mice.

Supplemental Figure 5. Plasma C3 in delFH-FHR and delFHR mice.

Supplemental Figure 6. Plasma C5 in delFH-FHR and delFHR mice.

Supplemental Figure 7. Mouse FHR proteins in delFH-FHR mice.

Supplemental Figure 8. Complement phenotype in hFH-FHR5 and hFH-FHR5mut mice

Supplemental Figure 9. Renal phenotype in hFH-FHR5 and hFH-FHR5mut mice.

Supplemental Figure 10. Genotype phenotype analysis in male FH-FHR-hFH-FHR5mut mice

Supplemental Table 1. Primers, guide RNAs and bridging repair oligonucleotides.

Supplemental Table 2. Renal Phenotyping in 12-month-old delFHR and delFH-FHR mice

Supplemental Table 3. Renal Phenotyping in 12-month-old hFH-FHR5 and hFH-FHR5mut mice

Supplemental Methods:

- Fluorescent In Situ Hybridization
- Zygote Injections

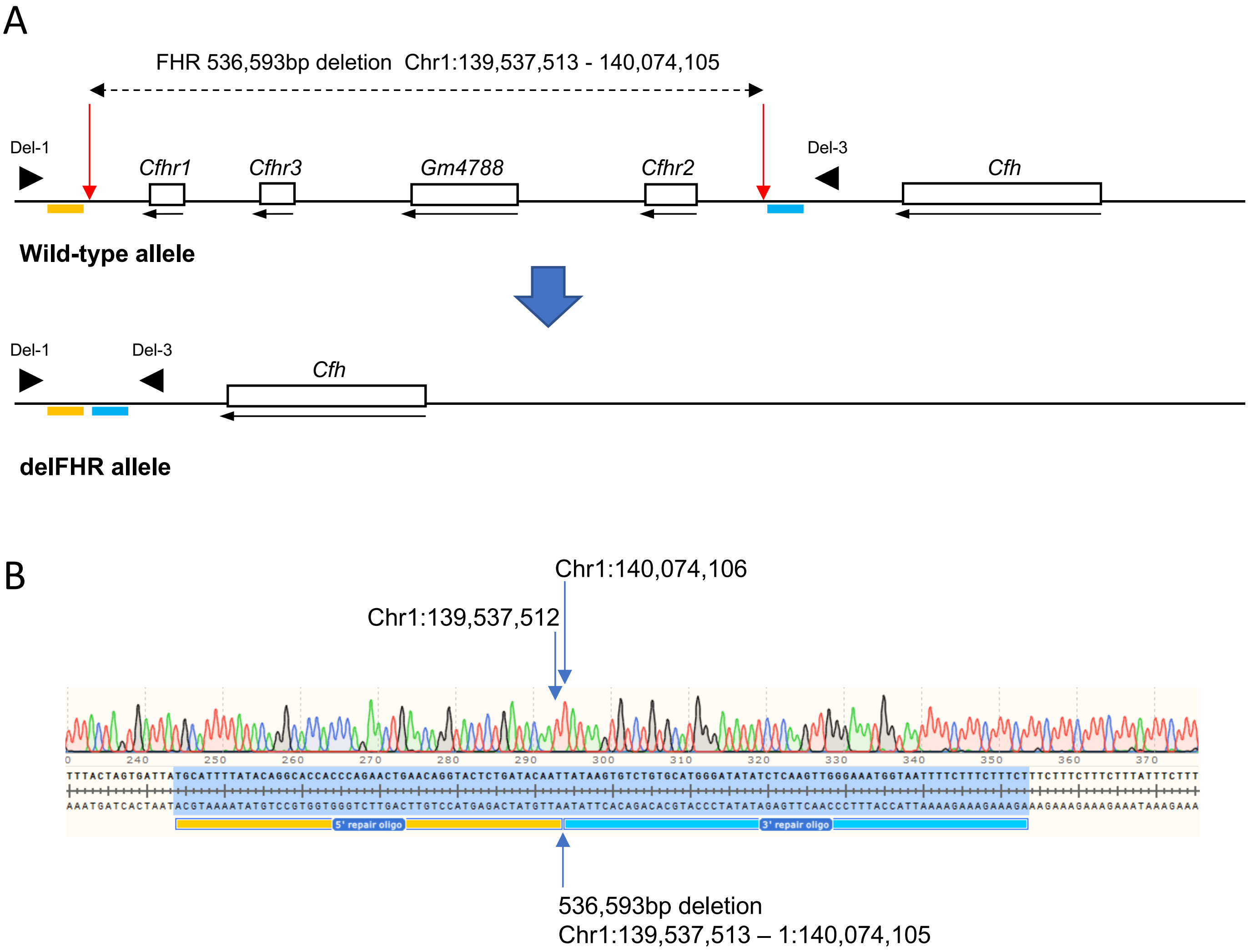

C

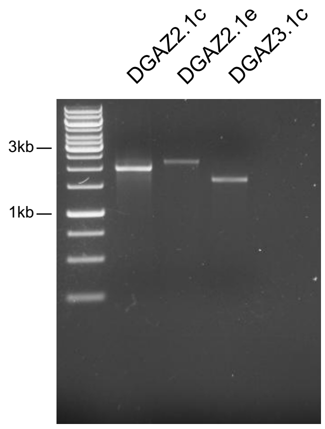

Mouse ID	Deletion (base pairs)	Deletion coordinates
DGAZ-2.1c	536,593	Chr1:139,537,513 – 1:140,074,105
DGAZ-2.1e	536,356	Chr1:139,537,668 – 1:140,074,023<br>T insertion at 1:139,537,520<br>G to A at 1:140,074,098<br>deletion TG at 1:140,074,099
DGAZ-3.1c	146,949	Chr1:139,537,670 – 1:139,684,618

Supplemental Figure 1. Generation of the delFHR allele. (A) Schematic showing the position of the guide RNAs (red arrows) and the bridging repair oligonucleotide (yellow/blue bars) that targets sequence flanking the desired deletion. Del-1 and Del-3 (black triangles) denote the primers used for identifying the deletion (see Supplementary Table 1). (B) Sequencing using these primers denoting that in this founder (DGAZ2.1c) the desired deletion has been achieved resulting in a deleted sequence of 536,593bp. Genome coordinates derived from Ensembl Mus Musculus version 100.38, Genome Reference Consortium Mouse Reference 38.p6. FHR – factor H-related.

A

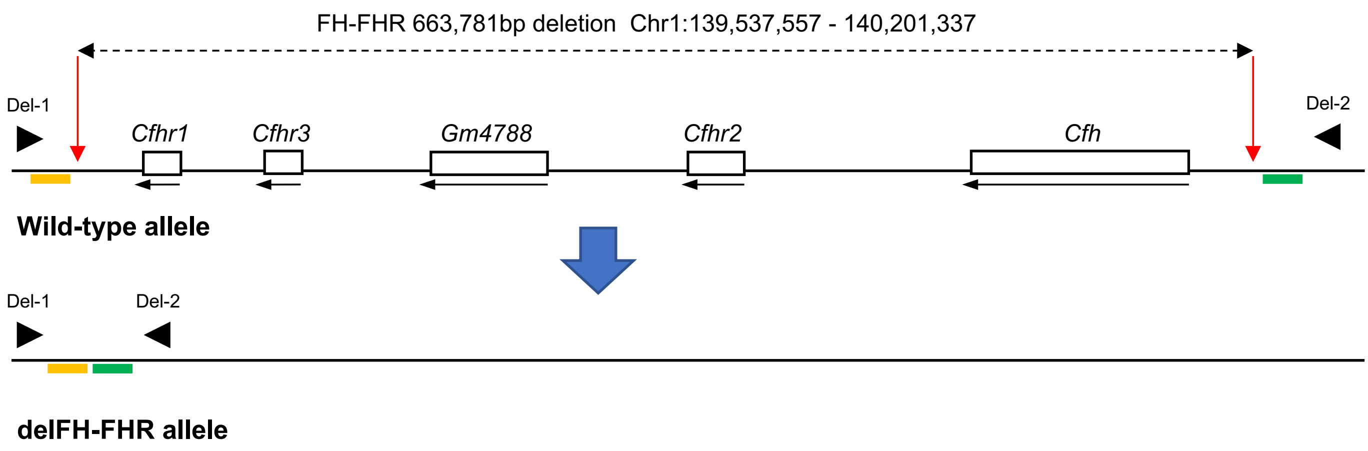

B

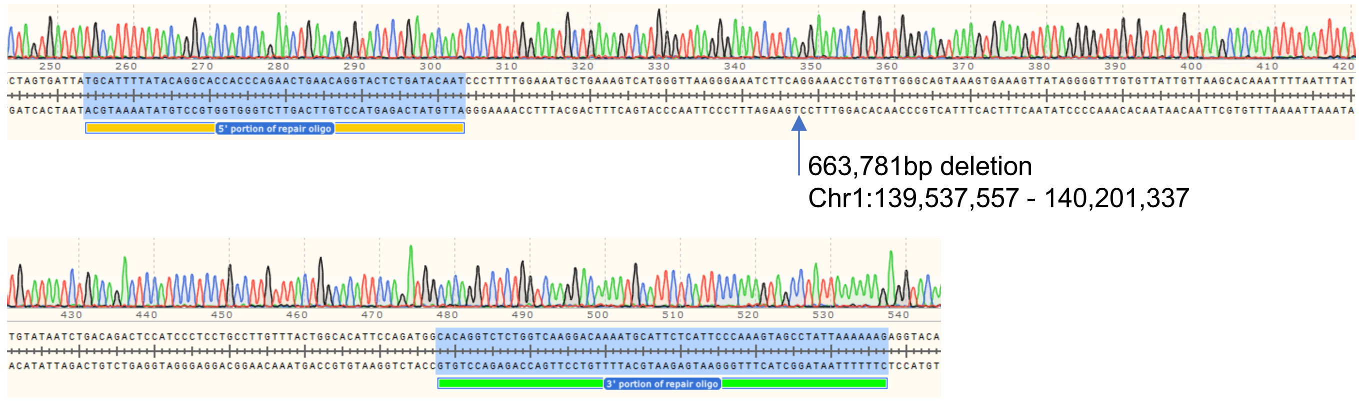

C

Mouse ID	Deletion (base pairs)	Deletion coordinates
DGCH-1.1b	664,620	Chr1:139,536,786 – 1:140,201,405<br>T insertion at 1:139,536,786
DGCH-4.1c	664,907	Chr1:139,537,066 – 1:140,201,972
DGCH-2.1b	663,826	Chr1:139,537,513 – 1:140,201,338<br>ACT insertion at 1:139,537,513
DGCH-2.1d	663,781	Chr1:139,537,557 – 1:140,201,337

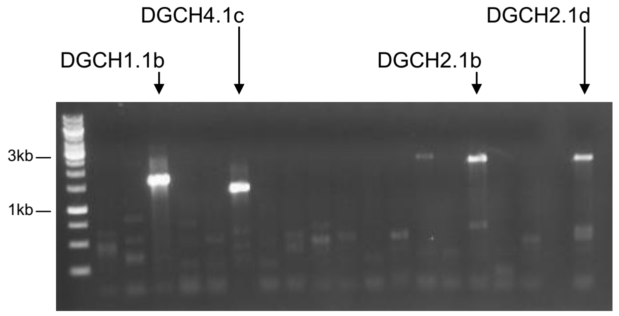

Supplemental Figure 2. Generation of the delFH-FHR allele. (A) Schematic showing the position of the guide RNAs (red arrows) and the bridging repair oligonucleotide (yellow/green bars) that targets sequence flanking the desired deletion. Del-1 and Del-2 (black triangles) denote the primers used for identifying the deletion (see Supplementary Table 1). (B) Sequencing using these primers denoting that in this founder the desired deletion has been achieved resulting in a deleted sequence of 663,780bp. Genome coordinates derived from Ensembl Mus Musculus version 100.38, Genome Reference Consortium Mouse Reference 38.p6. FHR – factor H-related, FH – Factor H.

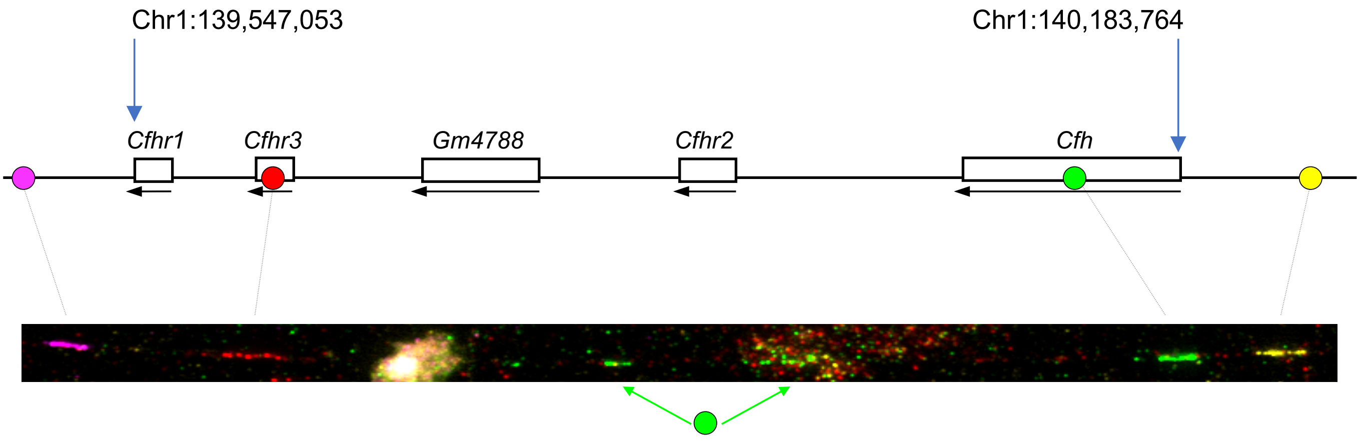

fibre FISH probe [Whitehead Institute ID]	Genomic coordinates
Probe 1 [WI1-1760L7]	1:139,454,716-1:139,497,830
Probe 2 [WI1-874H17]	1:139,600,720-1:139,634,562
Probe 3 [WI1-2805P14]	1:140,126,768-1:140,164,411
Probe 4 [WI1-277D21]	1:140,229,985-1:140,271,491

Gene	Genomic coordinates
Cfh	1:140,183,764 - 1:140,084,708
Cfhr2 (FHR-B)	1:139,804,167 - 1:139,858,718
Gm4788	1:139,697,623 - 1:139,781,243
Cfhr3	1:139,584,783 - 1:139,660,899
Cfhr1	1:139,547,053 - 1:139,560,272

Supplemental Figure 3. Fibre FISH probes across the FH-FHR region. Schematic showing the position of the four fibre FISH probes used. The image is from a wild-type litter mate control animal and demonstrates that all four probes bind in their expected locations. Additional signal for Probe 3 is seen in the centre of the locus and reflects the high degree of sequence similarity between this region and its genomic target. Table depicts Probe details and genomic coordinates. For reference the genomic coordinates of the *Cfh* and related genes are also shown. Genome coordinates derived from Ensembl Mus Musculus version 100.38, Genome Reference Consortium Mouse Reference 38.p6.

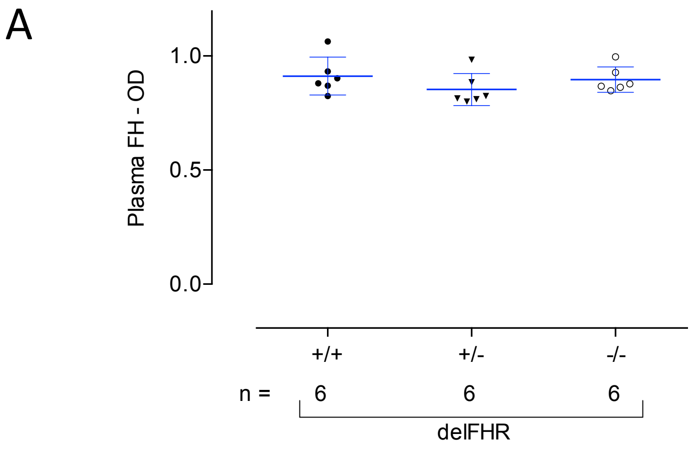

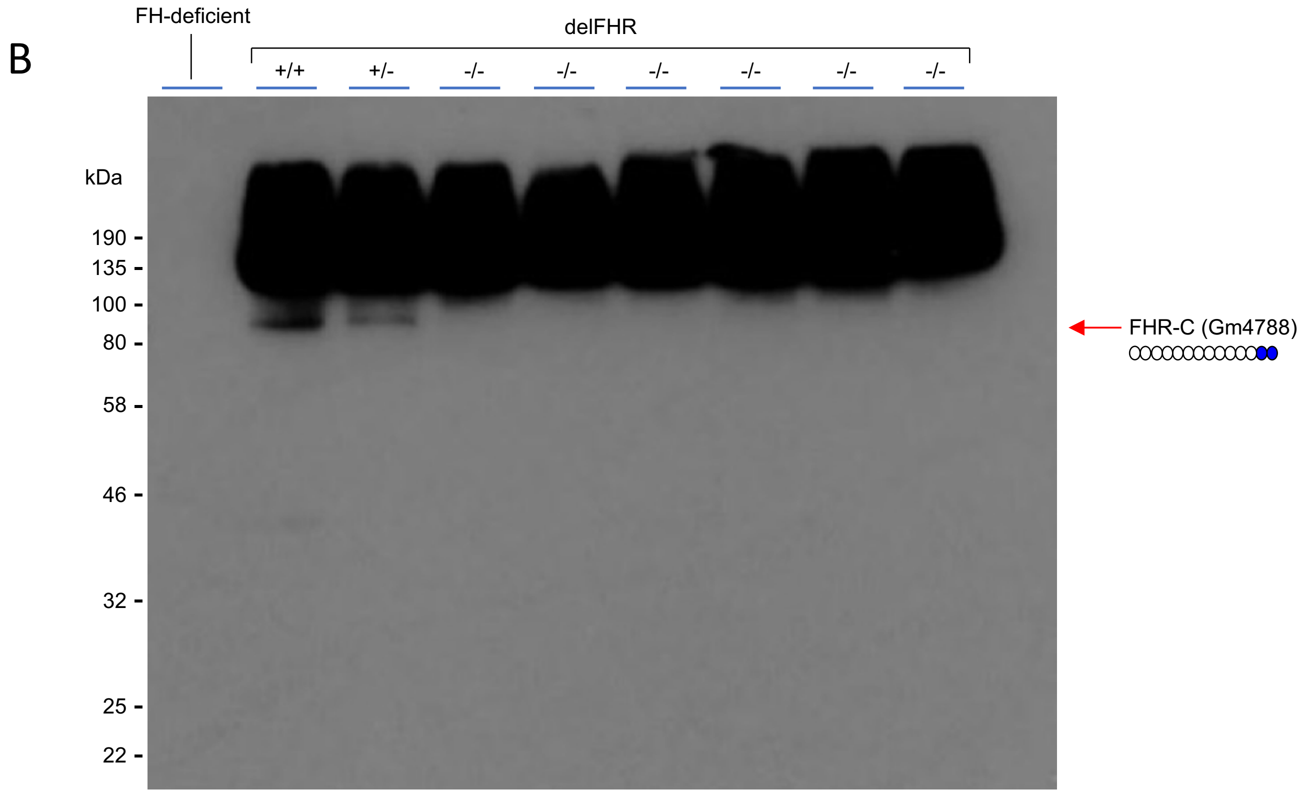

Supplemental Figure 4. Plasma FH and FHR-C in delFHR mice (A) Plasma FH levels by ELISA in delFHR mice. Bars denote mean and whiskers denote standard deviation. (B) Western blot of plasma FHR-C (arrows) in delFHR mice. Control lanes: plasma from FH-deficient mice. The FHR-C band is detectable in plasma from a delFHR wild-type litter mate, reduced in the plasma of a heterozygous delFHR animal and absent in homozygous delFHR samples. Primary antibody: Sheep anti-mouse FH (AF4999, R&D).

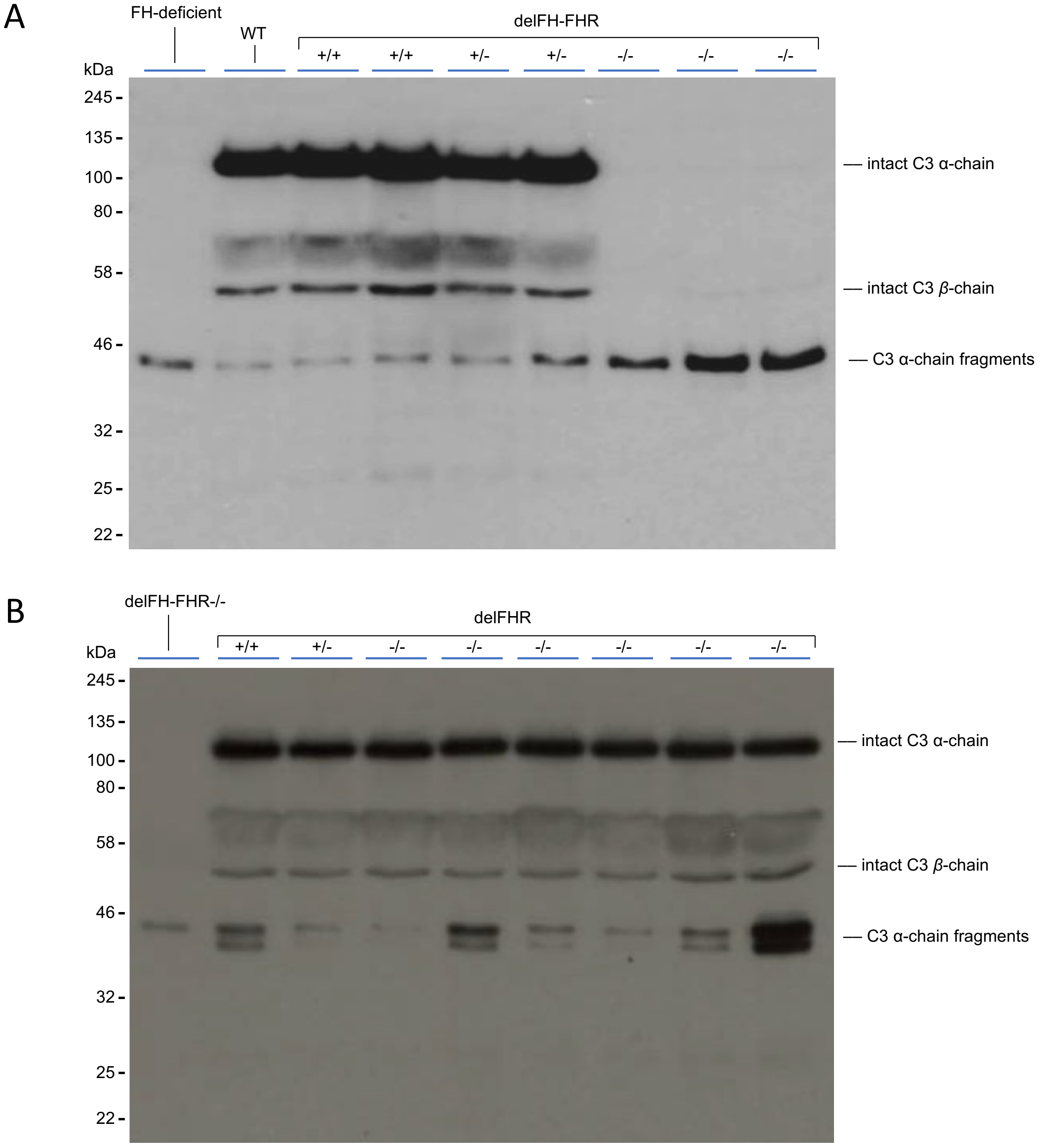

Supplemental Figure 5. Plasma C3 in delFH-FHR and delFHR mice (A) Western blot under reducing conditions of C3 from delFH-FHR plasma shows loss of the intact C3 α-chain band in homozygous mice comparable to that seen in plasma from FH-deficient mice (lane 1) and an increase in the C3 α-chain fragments. (B) Western blot under reducing conditions of C3 from delFHR plasma. All delFHR genotypes show the intact C3 α-chain band. Primary antibody: Goat anti-mouse C3 (#55444, MP Biomedicals).

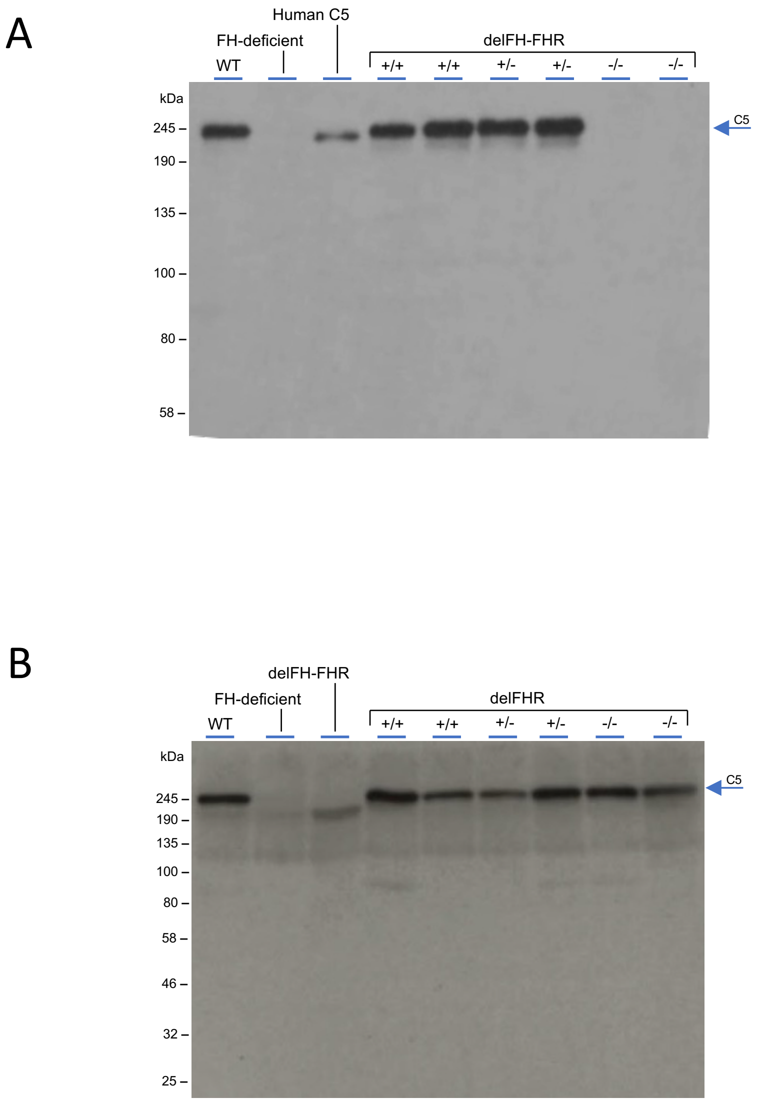

Supplemental Figure 6. Plasma C5 in delFH-FHR and delFHR mice. Western blot of plasma C5 in delFH-FHR (A) and delFHR mice (B). Control lanes: plasma from wild-type (WT), FH-deficient and delFH-FHR mice, and human C5 as indicated. The C5 band is absent in homozygous delFH-FHR mice and in the FH-deficient strain.

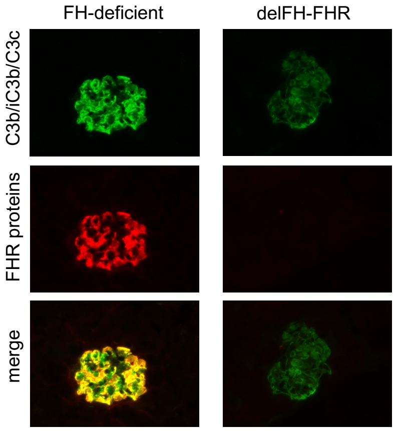

Supplemental Figure 7. Mouse FHR proteins in delFH-FHR mice. Glomerular co-staining for C3b/iC3b/C3c and FHR proteins. Abnormal glomerular FHR staining which co-localises with C3 is evident in the FH-deficient mice but absent in the delFH-FHR strain.

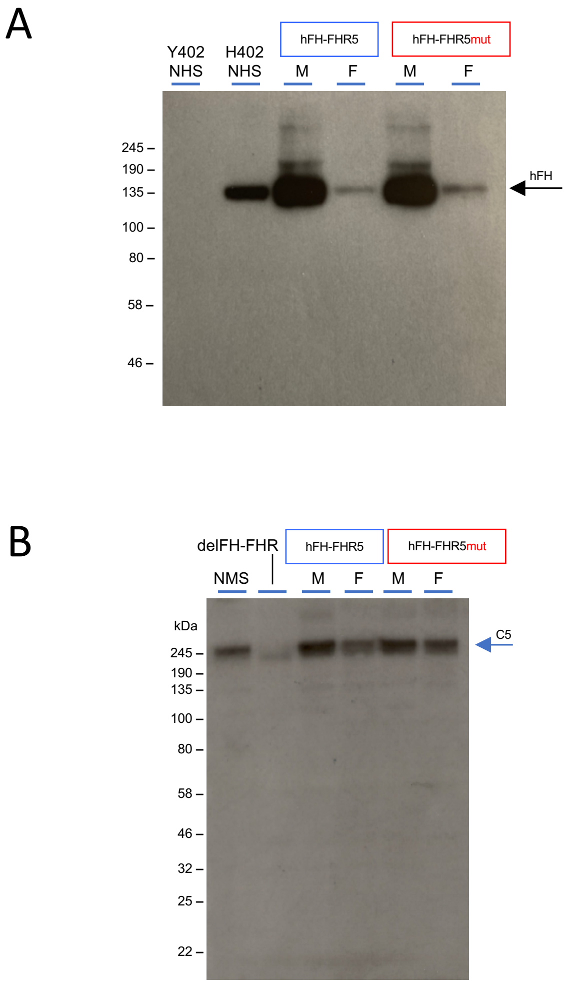

Supplemental Figure 8. Complement phenotype in hFH-FHR5 and hFH-FHR5mut mice. (A) Western blot of hFH demonstrating expression of H402-hFH. Control lanes: H402/Y402-specific human sera. (B) Western blot of plasma C5. Control lanes: NMS – normal mouse sera, delFH-FHR plasma.

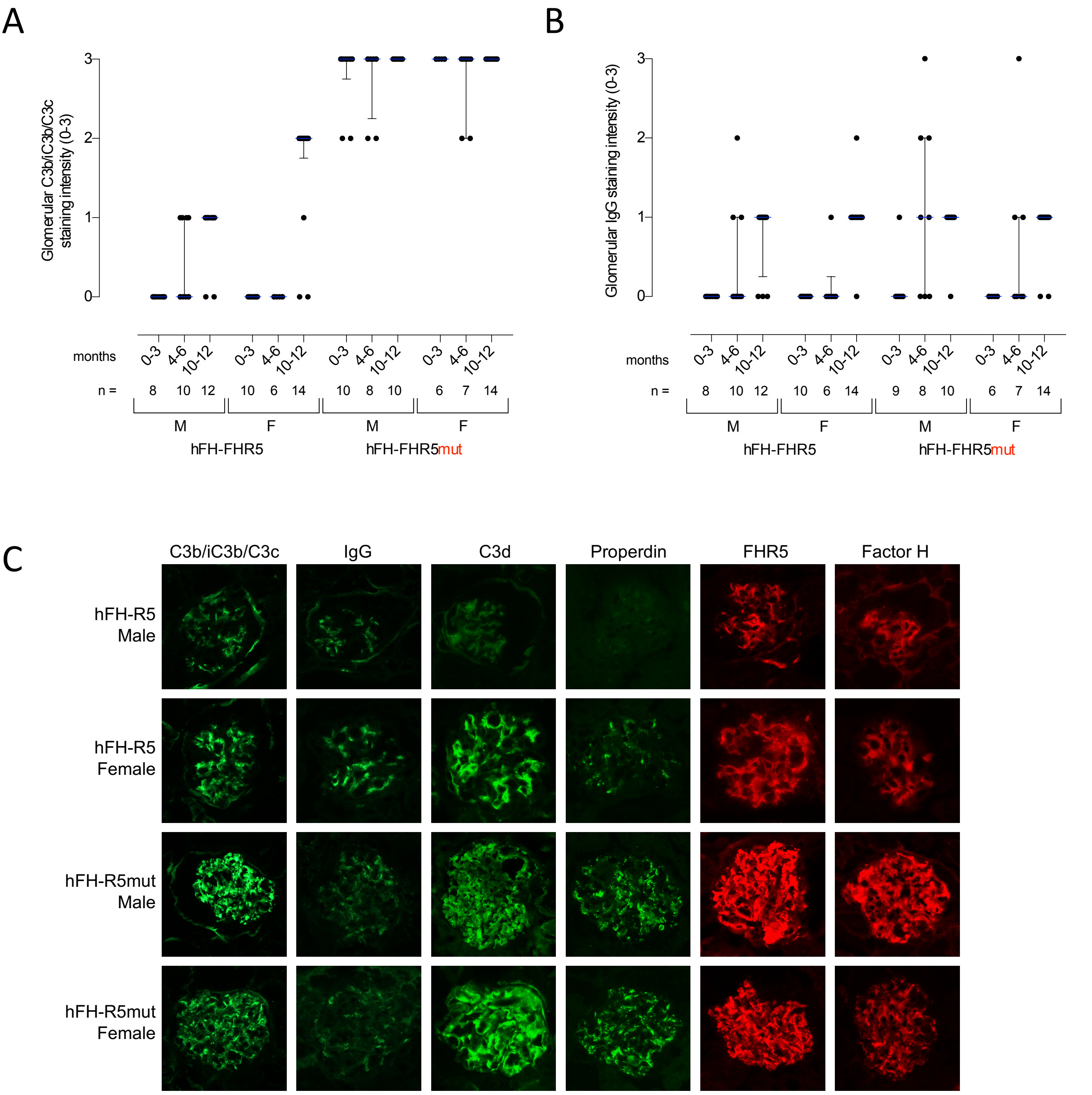

Supplemental Figure 9. Renal phenotype in hFH-FHR5 and hFH-FHR5mut mice. Glomerular C3b/C3b/C3c (A) and IgG (B) staining scores over time. Bars denote median and whiskers interquartile range. (C) Representative glomerular immunostaining images of C3b/iC3b/C3c, IgG, C3d, properdin, FHR5 and FH in 12-month-old hFH-FHR5 and hFH-FHR5mut mice.

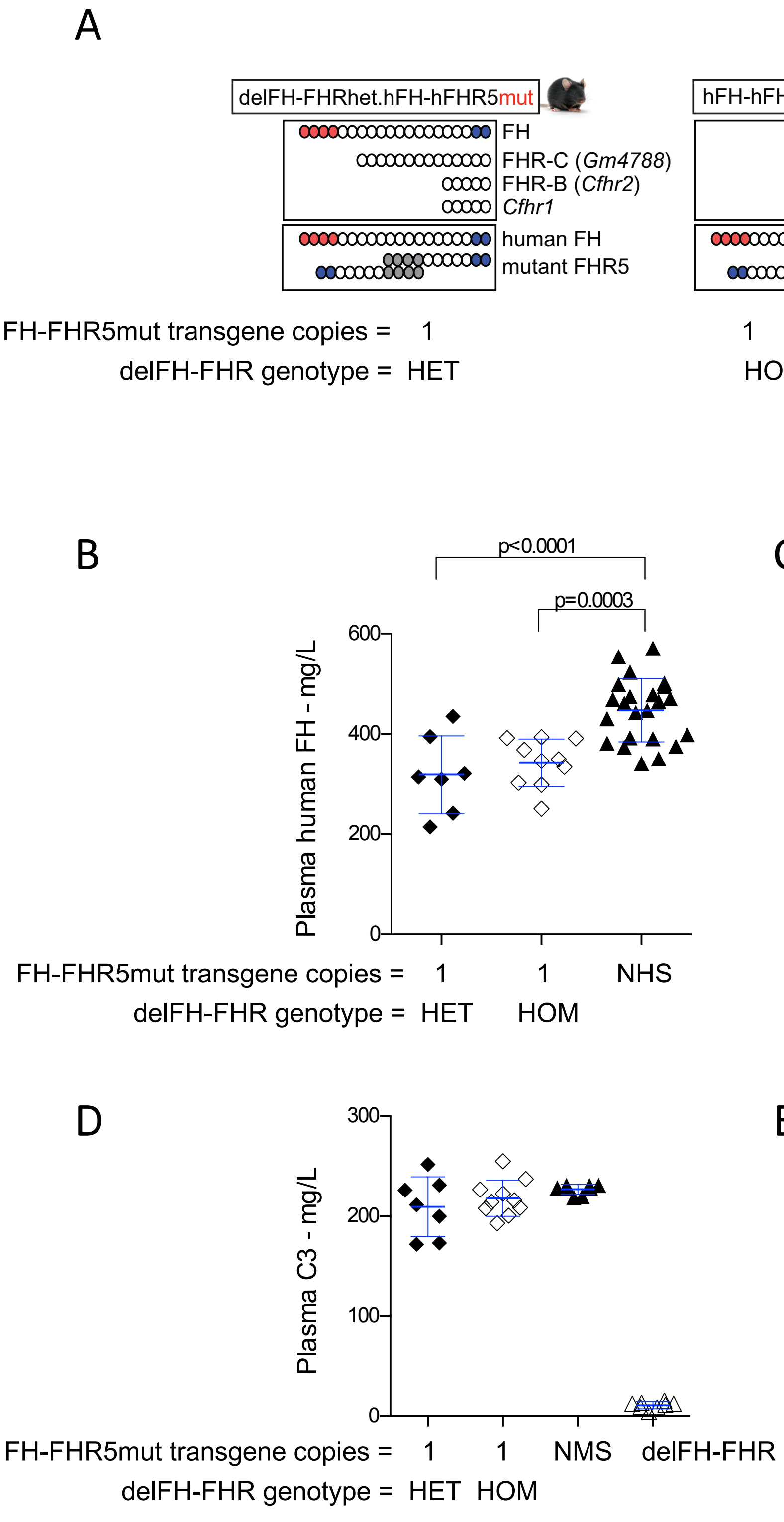

Supplemental Figure 10. Genotype phenotype analysis in male delFH-FHRhet.hFH-FHR5mut mice. (A) The delFH-FHRhet.hFH-FHR5mut male mice (n=7) are delFH-FHR heterozygotes and express one copy of the transgene. The hFH-FHR5mut mice (n=10) are delFH-FHR homozygotes and express one copy of the transgene. Schematic depicts the proteins present in the strains. FHR – Factor H-related; FH – factor H. Plasma FH (B), FHR5 (C) and C3 (D) levels by ELISA. Plasma hFH and FHR5mut levels were approximately half those seen with two copies of the transgenes. Plasma C3 levels were normal. Bars denote mean and whiskers denote standard deviation. P values derived from ANOVA with Bonferroni multiple comparisons test. NMS – normal mouse sera (n=8); NHS – normal human sera (n=15); HET – heterozygous; HOM – homozygous. (D) Glomerular C3b/iC3b/C3c staining scores. Bars denote median and whiskers interquartile range.

	DelFHR deletion	DelFH-FHR deletion
Guide RNAs	5' guide RNA-1<br>GCTTATTTATTGGTAGGATGGGG<br>1:139,537,652 - 139,537,675<br><br>5' guide RNA-2<br>GACTTTCAGCATTTCCAAAAGGG<br>1:139,537,512 - 139,537,535<br><br>3' guide RNA-delFHR-1<br>TGCTAGTAGAGTCTCTCAGAAGG<br>1:140,074,027 - 140,074,049<br><br>3' guide RNA-delFHR-2<br>TTAGTATCAGCAATAGTGTAAGG<br>1:140,074,083 - 140,074,105	5' guide RNA-1<br>GCTTATTTATTGGTAGGATGGGG<br>1:139,537,652 - 139,537,675<br><br>5' guide RNA-2<br>GACTTTCAGCATTTCCAAAAGGG<br>1:139,537,512 - 139,537,535<br><br>3' guide RNA-delFH-FHR-1<br>AAGAACTATGCTTTATAGCGAGG<br>1:140,201,299 - 140,201,321<br><br>3' guide RNA-delFH-FHR-2<br>CGAGGATACTGAATATATCCAGG<br>1:140,201,317 - 140,201,339
Bridging Repair oligonucleotide	TGCATTTTATACAGGCACCACCCAGAACTGAACAG<br>GTACTCTGATACAATTATAAGTGTCTGTGCATGGGA<br>TATATCTCAAGTTGGGAAATGGTAATTTTCTTTCTTT<br>CT<br>1:139,537,463 - 139,537,512 [yellow]; 1:140,074,106 - 140,074,165 [blue]	TGCATTTTATACAGGCACCACCCAGAACTGAACAG<br>GTACTCTGATACAATCACAGGTCTCTGGTCAAGGA<br>CAAAATGCATTCTCATTCCCAAAGTAGCCTATTAAA<br>AAAG<br>1:139,537,463 - 139,537,512 [yellow]; 1:140,201,468 - 140,201,527 [blue]
Primers used to identify deletions	Del-1<br>CTCAAGGGCAGCAGTAAACC<br>1:139,536,177 - 139,536,196<br><br>Del-3<br>TTCATGGTCCCACTGAAACA<br>1:140,074,730 - 140,074,749<br><br>amplicon - 1980bp	Del-1<br>CTCAAGGGCAGCAGTAAACC<br>1:139,536,177 - 139,536,196<br><br>Del-2<br>GGGTCCTTTCAACACAGTGC<br>1:140,202,444 - 140,202,463<br><br>amplicon - 2332bp
Genotyping Primers	Wild-type allele at 5' end of locus<br><br>5F1<br>TGGGGAGCACATATGAGACA<br>1:139,537,186 - 139,537,205<br><br>5R1<br>ATGGGGCATTGATTATTGGA<br>1:139,537,836 - 139,537,817<br><br>amplicon - 651bp	Wild-type allele at 5' end of locus<br><br>5F1<br>TGGGGAGCACATATGAGACA<br>1:139,537,186 - 139,537,205<br><br>5R1<br>ATGGGGCATTGATTATTGGA<br>1:139,537,836 - 139,537,817<br><br>amplicon - 651bp
	Wild-type allele at 3' end of locus<br><br>3SF1<br>CCAGAAGTGGGGTTAGTGAAG<br>1:140,073,755 - 140,073,775<br><br>3SR1<br>TTCATGGTCCCACTGAAACA<br>1:140,074,749 - 140,074,730<br><br>amplicon - 995bp	Wild-type allele at 3' end of locus<br><br>3LF1<br>GTGGGTATGGGGGTCTTTTG<br>1:140,201,027 - 140,201,046<br><br>3LR1<br>GCCAGTAAACAAGGCAGGAG<br>1:140,201,454 - 140,201,435<br><br>amplicon - 428bp
	DelSF1<br>TCCTGAAGGCTGGAACAAGT<br>1:139,537,300 - 139,537,319<br><br>DelSR1<br>GTTGGGAGGGGATATGGAAT<br>1:140,074,328 - 140,074,309<br><br>amplicon - 436bp	DelLF1<br>TCCTGAAGGCTGGAACAAGT<br>1:139,537,300 - 139,537,319<br><br>DelLR1<br>TAAACAAGGCAGGAGGGATG<br>1:140,201,449 - 140,201,430<br><br>amplicon – 368bp

Supplemental Table 1. Primers, guide RNAs and bridging repair oligonucleotides. The first 50 nucleotides (yellow highlight) of both repair oligonucleotides target genomic location 1:139,537,463 – 1:139, 537, 512 which is downstream from the *Cfhr1* gene. For the delFHR repair oligonucleotide the remaining 60 nucleotides (blue highlight) target genomic location 1:140,074,106 – 1:140,074,165, an intergenic region between the *Cfhr2* and *Cfh* genes. For the delFH-FHR repair oligonucleotide the remaining 60 nucleotides (green highlight) target genomic location 1:140,201,468 – 1:140,201,527 upstream of the *Cfh* gene. All primer sequences are denoted 5' to 3'. Genome coordinates derived from Ensembl Mus Musculus version 100.38, Genome Reference Consortium Mouse Reference 38.p6

Genotype	delFHR	Wild-type[a]	delFH-FHR	FH-deficient
Age (months)	8	8	8	11
Number (M/F)	57 (35/22)	11 (8/3)	30 (17/13)	14 (5/9)
Plasma C3[b] – mg/L	186.1(26.3)	190 (16.2)	9.9 (6.2)	7.4 (3.3)
Serum urea[b] – mmol/L	6 (3.2)	7.8 (1.6)	13.1 (2.7)	13.9 (2.2)
Dipstick proteinuria ++ or more	0	0	0	0
Dipstick haematuria	0	0	0	0
Glomerular cellularity[c] (0-4)	0 (0-0)	0 (0-0)	2 (1-3)	1 (0-2)
Capillary wall thickening[c] (0-10)	0 (0-0)	0 (0-0)	0.5 (0-1)	0 (0-1)
Segmental sclerosis[c] (0-1)	0 (0-0)	0 (0-0)	1 (0-1)	1 (0-1.25)
Mesangial expansion[c] (0-1)	0 (0-1)	0 (0-1)	1 (0-1)	0.5 (0-1)
Glomerular C3b/iC3b/C3c staining pattern Normal/Linear (% affected)	Normal (100%)	Normal (100%)	Linear (100%)	Linear (100%)
Tubulo-interstitial C3b/iC3b/C3c staining Present/Absent (% affected)	Present (100%)	Present (100%)	Absent (100%)	Absent (100%)

[a]Wild-type litter-mates of the deFHR strain;

[b]Values represent mean (standard deviation);

[c]Values represent median (interquartile range, IQR).

Supplemental Table 2. Renal Phenotyping in 8-month-old delFHR and delFH-FHR mice

Genotype	hFH-FHR5		hFH-FHR5mut	
Complement Profile				
Sex (number)	M (n=30)	F (n=31)	M (n=30)	F (n=29)
Plasma Factor H[a] – mg/L[1]	1121 (70.4)[3, 4]	140.2 (42.9)	826.3 (130.7)[3]	111.3 (55.6)
Plasma Factor H-related 5[a] – mcg/ml[2]	13.7 (1.3)[3]	0.8 (0.5)	13.5 (1.6)[3]	0.8 (0.5)
Plasma C3[a] – mg/L	208.9 (20.5)[3, 5]	87 (22.4)	180.3 (43.6)[3]	76.6 (32.5)
Renal phenotyping at 12 months of age				
Sex (number)	M (n=12)	F (n=10)	M (n=10)	F (n=10)
Serum urea[a] – mmol/L	12.6 (1.5)	14.2 (1.3)	12.9 (1.4)	12.8 (1.4)
Serum albumin[a] – g/dL	4.6 (0.9)	4.6 (1)	4.6 (0.8)	4.9 (1)
Dipstick proteinuria ++ or more	0	0	0	0
Dipstick haematuria	0	0	0	0
Glomerular cellularity[b] (0-4)	0 (0-0)	0 (0-0)	0 (0-0)	0 (0-0.25)
Capillary wall thickening[b] (0-10)	0 (0-0)	0 (0-0)	7.5[6] (6-10)	0 (0-0)
Segmental sclerosis[b] (0-1)	0 (0-0.75)	0 (0-0)	0 (0-0)	0 (0-0.25)
Mesangial expansion[b] (0-1)	0 (0-1)	0 (0-0)	0 (0-1)	0 (0-0)
Glomerular C3b/iC3b/C3c staining[b] (0-3)	1 (1-1)	2 (2-2)	3[6] (3-3)	3 (3-3)
Glomerular C3d staining[b] (0-3)	1 (1-1)	2 (2-2)	3[6] (3-3)	3 (3-3)
Glomerular IgG staining[b] (0-3)	1 (0.25-1)	1 (1-1)	1 (1-1)	1 (0-1)
Glomerular Properdin staining[b] (0-3)	0 (0-0)	1 (1-1)	3[6] (3-3)	3 (3-3)
Glomerular FH staining intensity[b] (0-3)	1 (1-1)	2 (2-2)	3[6] (3-3)	3 (3-3)
Glomerular FHR5 staining intensity[b] (0-3)	1 (1-1)	2 (2-2)	3[6] (3-3)	3 (3-3)
Glomerular CD68 staining[b]	2 (1.7-2.3)	2.7 (2.1-2.9)	3.5[7] (2.6-4.2)	4.7 (2.7-6)

[a]Values represent mean (standard deviation) and P values derived from ANOVA with Bonferroni Multiple Comparison Test;

[b]Values represent median (interquartile range, IQR) and P values derived from Kruskal-Wallis with Dunn's Multiple Comparison Test;

[1]Mean human FH plasma concentration using this assay = 447.2 mg/L (SD 63.3, n=23);

[2]Mean human FHR5 plasma concentration using this assay = 6.6 mcg/ml (SD 1.1, n=15);

[3]$p<0.0001$ vs female and [4]$p<0.0001$ and [5]$p<0.0021$ vs male mice;

[6]$p\leq0.0001$ and [7]$p=0.0392$ vs male hFH-FHR5 mice.

Supplemental Table 3. Renal Phenotyping in 12-month-old hFH-FHR5 and hFH-FHR5mut mice

Supplemental Methods

Fluorescent In Situ Hybridization. Fiber-FISH performed as described previously: Boroviak K, Doe B, Banerjee R, Yang F, and Bradley A. Chromosome engineering in zygotes with CRISPR/Cas9. Genesis. 2016;54(2):78-85. Briefly, extended chromatin and DNA fibres were prepared by alkaline lysis from mouse splenocytes. Fosmid DNA was labelled using biotin-16-dUTP, digoxigenin-11-dUTP, dinitrophenol (DNP)-11-dUTP (Jena Bioscience). Biotin labelled probe was detected with Cy3-streptavidin (Sigma-Aldrich); Digoxigenin-labelled probe was detected with monoclonal mouse anti-DIG IgG (Sigma-Aldrich) and Texas red conjugated donkey anti-mouse IgG (Invitrogen); DNP-labelled probe was detected with rabbit anti-DNP and Alexa 488 conjugated goat anti-rabbit IgG. After detection, slides were mounted with SlowFade Diamond® (Invitrogen) mounting solution containing 4′,6-diamidino-2-phenylindole (Invitrogen). FISH images were captured and processed using the SmartCapture (Digital Scientific, UK) digital imaging system using a Zeiss microscope (Axioplan 2 Imaging or AxioImager, DI) equipped with narrow bandpass filters for Cy5, Cy3, Texas Red, FITC and DAPI fluorescence and a cooled CCD camera (Hamamatsu).

Zygote Injections. Four- to five-week-old C57BL/6NTac females were super-ovulated by intra-peritoneal (IP) injection of 5 IU of pregnant mare's serum (PMSG) at 12:00 to 13.00 hours (on a 12 hour light/dark cycle, on at 07:00/off at 19:00) followed 48 hours later by an IP injection of 5 IU human chorionic gonadotrophin (hCG) and mated overnight with C57BL/6NTac stud males. The next morning the females were checked for the presence of a vaginal copulation plug as evidence of successful mating, oviducts were dissected at approximately 21–22 hours post hCG and cumulus oocyte complexes from these were released and treated with hyaluronidase as previously described: Perry GH, Yang F, Marques-Bonet T, Murphy C, Fitzgerald T, Lee AS, et al. Copy number variation and evolution in humans and chimpanzees. Genome Res. 2008;18(11):1698-710. Fertilized 1-cell embryos were selected and maintained at 37°C in KSOM media prior to cytoplasmic injection. Injections were carried out between 24 and 27 hours post hCG. About 50 ng/μL Cas9 mRNA, 25 ng/μL gRNA (each) and 100 ng/μL oligonucleotide were mixed in RNase free water, backfilled into an injection needle with positive balancing pressure and injected into the cytoplasm of fertilized 1-cell embryos held in FHM medium. A successful injection was indicated by visible movement in the cytoplasm. Injected embryos were briefly cultured and viable embryos were transferred the same day by oviductal embryo transfer into a 0.5 days post-coital pseudo-pregnant female F1 (CBA/C57BL/6J) recipients.
